# Widespread turnover of a conserved cis*-*regulatory code across 589 grass species

**DOI:** 10.1093/molbev/msaf324

**Published:** 2025-12-10

**Authors:** Charles O Hale, Sheng-Kai Hsu, Jingjing Zhai, Aimee Schulz, Taylor Aubuchon-Elder, Germano Costa-Neto, Allen Gelfond, Mohamed Z El-Walid, Matthew Hufford, Elizabeth A Kellogg, Thuy La, Alexandre P Marand, Arun S Seetharam, Armin Scheben, Michelle C Stitzer, Travis Wrightsman, Maria Cinta Romay, Edward S Buckler

**Affiliations:** Section of Plant Breeding and Genetics, Cornell University, Ithaca, NY 14853 USA; Institute for Genomic Diversity, Cornell University, Ithaca, NY 14853 USA; Institute for Genomic Diversity, Cornell University, Ithaca, NY 14853 USA; Section of Plant Breeding and Genetics, Cornell University, Ithaca, NY 14853 USA; Donald Danforth Plant Science Center, St. Louis, MO 63132 USA; Institute for Genomic Diversity, Cornell University, Ithaca, NY 14853 USA; Section of Plant Breeding and Genetics, Cornell University, Ithaca, NY 14853 USA; Section of Plant Breeding and Genetics, Cornell University, Ithaca, NY 14853 USA; Department of Ecology, Evolution, and Organismal Biology, Iowa State University, Ames, IA 50011 USA; Donald Danforth Plant Science Center, St. Louis, MO 63132 USA; Institute for Genomic Diversity, Cornell University, Ithaca, NY 14853 USA; Department of Genetics, University of Michigan, Ann Arbor, MI 48109 USA; Department of Ecology, Evolution, and Organismal Biology, Iowa State University, Ames, IA 50011 USA; Simons Center for Quantitative Biology, Cold Spring Harbor Laboratory, Cold Spring Harbor, NY 11724 USA; Institute for Genomic Diversity, Cornell University, Ithaca, NY 14853 USA; Section of Plant Breeding and Genetics, Cornell University, Ithaca, NY 14853 USA; Institute for Genomic Diversity, Cornell University, Ithaca, NY 14853 USA; Section of Plant Breeding and Genetics, Cornell University, Ithaca, NY 14853 USA; Institute for Genomic Diversity, Cornell University, Ithaca, NY 14853 USA; USDA-ARS, Robert W. Holley Center for Agriculture and Health, Ithaca, NY 14853, USA

**Keywords:** regulatory evolution, plants, comparative genomics, cis-regulation

## Abstract

The growing availability of genomes from non-model organisms offers new opportunities to identify functional loci underlying trait variation through comparative genomics. While cis-regulatory regions drive much of phenotypic evolution, linking them to specific functions remains challenging. We identified 514 cis-regulatory motifs enriched in regulatory regions of five diverse grass species, with 73% consistently enriched across all, suggesting a deeply conserved regulatory code. Leveraging 57 new contig-level genome assemblies, we then quantified shared occupancy of specific motif instances within gene-proximal regions across 589 grass species, revealing widespread gain and loss over evolutionary time. Shared occupancy declined rapidly over the first few million years of divergence, yet ∼50% of motif instances were shared back to the origin of grasses ∼100 million years ago. We used phylogenetic mixed models to identify motif gains and losses associated with ecological niche transitions. Our models revealed significant environmental associations across 1282 motif–orthogroup combinations, including convergent gains of HSF/GARP motifs at an alpha-N-acetylglucosaminidase gene associated with occurrence in temperate environments. Our findings support a “stable motifs, variable binding sites” model in which cis-regulatory evolution involves turnover of thousands of individual binding site instances while largely preserving transcription factors’ binding preferences. Our results highlight the potential of comparative genomics and phylogenetic mixed models to reveal the genetic basis of complex traits.

## Introduction

Cis-regulatory changes are arguably the most important genetic mechanism of evolutionary innovation, particularly over relatively shallow evolutionary time scales (King and Wilson 1975; Carroll 2008). While the trans-acting regulators such as transcription factors (TFs) typically have widespread effects genome-wide (Signor and Nuzhdin 2018), the evolution of cis-regulatory regions enables finely tuned gene expression evolution at individual genes (Wittkopp et al. 2004; Prud’homme et al. 2007; Wray 2007). Variation within noncoding regions explains roughly half of additive trait variance within maize populations with much of this attributable to open chromatin regions found within 1 kbp of genes (Rodgers-Melnick et al. 2016), underscoring the importance of gene-proximal regulatory regions in driving phenotypic evolution within species.

TF binding sites (TFBS) are key components of noncoding regulatory regions (Schmitz et al. 2021). TFBS are typically composed of a core DNA sequence motif that is recognized and bound by its cognate TF. In plants, TFBS variants have been shown to be associated with the evolution of traits including inflorescence branching (Hendelman et al. 2021), abiotic stress tolerance (Jiang et al. 2022; Zeng et al. 2025), and fruit shape (Hu et al. 2024). Variants in the 5′ UTR and proximal promoter region are often particularly important for modulating expression levels (Cui et al. 2023; Voichek et al. 2024). Despite the importance of these TF binding variants in driving phenotypic evolution, characterizing key variants remains a major challenge. TF binding assays such as Chromatin Immunoprecipitation Sequencing (ChIP-seq), MNase-defined cistrome-Occupancy Analysis (MOA-seq), and DNA affinity purification sequencing (DAP-seq) have allowed precise characterization of where TFs bind throughout plant genomes (O’Malley et al. 2016; Savadel et al. 2021; Marand et al. 2023), but adapting them to work in non-model systems can be difficult. In particular, the increasing number of sequenced taxa requires scalable methods to discover key cis-regulatory features.

Characterizing evolutionary constraint and convergence across large numbers of taxa has emerged as a powerful strategy for determining genetic function from DNA sequence alone (Smith et al. 2020). The identification of conserved noncoding sequences has been used to identify highly important cis-regulatory features across non-model taxa (Haudry et al. 2013; Partha et al. 2017; Song et al. 2021; Stitzer et al. 2025). A limitation of this approach, however, is that many key cis-regulatory regions cannot be aligned reliably (Schmidt et al. 2010; Phan et al. 2025). Additionally, the extent of nucleotide-level conservation does not reliably predict the degree of shared function (Yang et al. 2015; Wong et al. 2020). Nucleotide-level-conservation-free characterization approaches can offer a broader view of cis-regulatory evolution (Kaplow et al. 2022, 2023).

Due to these advances, our understanding of cis-regulatory evolution and its phenotypic implications is rapidly increasing. Still, some of the fundamental principles of regulatory evolution remain incompletely understood. A growing body of evidence indicates that TFs and their binding site preferences remain largely intact within lineages, suggesting a conserved “regulatory code” (Stergachis et al. 2014; Nitta et al. 2015; Tu et al. 2020). Changes to the regulatory code can occur via expansions or contractions of TF families that lead to the birth or extinction of particular TF binding preferences (Lehti-Shiu et al. 2017). Across deep evolutionary divergence, such as between plants and animals, widespread differences are observed between TFs and their binding preferences (Riechmann et al. 2000). The field lacks a clear consensus on the extent to which changes to TF binding preferences occur over varying time scales, as well as how and when changes occur. A deeper understanding of regulatory code evolution will inform how to effectively transfer genetics across species.

Another emerging model of regulatory evolution is that while TF binding preferences tend to be deeply conserved, individual instances of TFBS are extensively gained and lost over evolutionary time. We refer to this as the “stable motifs, variable binding sites” hypothesis. Previous studies have estimated that only 20% of TFBS instances are conserved between humans and mice (Stergachis et al. 2014), and 20% to 40% of GOLDEN2-LIKE binding sites were found to be conserved between maize and rice (Tu et al. 2022). Relatively few studies so far have traced TFBS evolution across a large number of taxa (but see Andrews et al. (2023)), or associated particular gain and loss events with diversification and adaptation at the macroevolutionary scale.

Grasses (Poaceae) have diversified widely over roughly 100 million years of evolution (Gallaher et al. 2022), occupying habitats ranging from tropical savannas to the Arctic tundra (Bouchenak-Khelladi et al. 2010; Edwards and Smith 2010; Spriggs et al. 2014; Lehmann et al. 2019). Grasses’ ecological diversification has been intertwined with repeated innovations in life history strategies (annual vs. perennial) (Kellogg 2015), photosynthetic pathway transitions (Grass Phylogeny Working Group II 2012), and photoperiod changes (Fjellheim et al. 2014). Despite these significant shifts, aspects of genome structure such as gene content and collinearity are largely preserved across grass lineages (Bennetzen and Freeling 1993; McSteen and Kellogg 2022; Mascher et al. 2024), although ploidy (Stebbins 1985; Zhang et al. 2024), genome size (Bennett and Smith 1976), and gene regulatory patterns (Meng et al. 2021; Stitzer et al. 2025) vary widely across the family. Comparative genomic analyses across grasses are therefore well-suited to illuminate how broad and repeated environmental adaptation unfolds in complex genomes (Buell 2009).

We hypothesized that gain/loss of many individual TFBS across the genome—rather than changes at a few master regulators—underlies grass diversification, enabling grasses to fine-tune gene regulation while maintaining regulatory logic. To test this hypothesis, we performed large-scale comparative genomic analyses across a collection of 727 genome assemblies representing 589 grass species. After establishing that grasses share a conserved set of TF motifs enriched in cis-regulatory regions, we quantified gain and loss of motif instances within gene-proximal regions (500 bp or 1 kbp upstream of aligned translation start sites). Using this set of motif instances, we performed association mapping across species to identify examples of motif gain and loss associated with environmental variables. We documented widespread gain and loss of motif instances as grasses diversified. Additionally, we revealed 1282 environmentally associated motif gain/loss instances with moderate to weak levels of convergence. Together, our findings support a model of cis-regulatory evolution in which adaptation proceeds over macroevolutionary time scales via hundreds to thousands of cis*-*regulatory changes at small-effect downstream genes rather than at a few large-effect regulators.

## Results

### Grasses share a deeply conserved **cis*-***regulatory code

**Figure 1:**
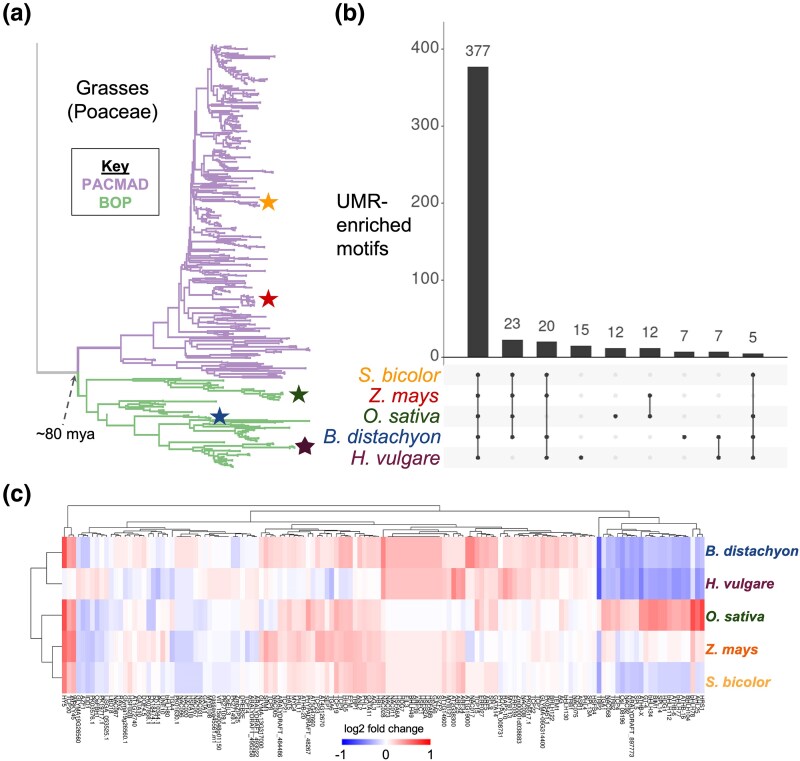
Grasses share a conserved set of UMR-enriched motifs. a) Phylogenetic tree of 589 Poaceae species. Five representative species are starred: *S. bicolor* (orange), *Z. mays* (red), *O. sativa* (dark green), *B. distachyon* (blue), and *H. vulgare* (dark purple). b) Enrichment of TF motifs in UMRs across five representative grass species. The intersection bars show the number of enriched motifs for each species set. Intersections with fewer than five motifs are not shown. c) Log2 fold change UMR enrichments for 114 motifs with varied enrichment patterns. Rows and columns are clustered by enrichment similarity.

We hypothesized that a highly similar repertoire of motifs are enriched in cis-regulatory regions across grasses. We estimated cis-regulatory regions using unmethylated regions (UMRs) available from five species (*Brachypodium distachyon*, *Oryza sativa*, *Sorghum bicolor*, *Zea mays*, and *Hordeum vulgare*). These five species last shared a common ancestor before the BOP (Bambusoideae, Oryzoideae, and Pooideae) and PACMAD (Panicoideae, Aristidoideae, Chloridoideae, Micrairoides, Arundinoideae, and Danthonioideae) clades diverged approximately 80 million years ago (Gallaher et al. 2022) (Fig. 1a). UMRs stably mark functional regulatory and genic regions of plant genomes and are rich in TFBS (Crisp et al. 2020). To determine which cis-regulatory motifs are commonly enriched in regulatory regions, we quantified UMR enrichment of 704 experimentally derived plant TF binding motifs from the JASPAR 2024 database (Rauluseviciute et al. 2024). Using randomized UMR sequences with preserved dinucleotide frequencies as background, we measured enrichment of each JASPAR motif in true UMRs relative to dinucleotide-shuffled UMRs. One caveat to this approach is that due to the high sequence similarity among motifs within some TF families, similar motifs cannot be reliably distinguished and may be “double counted” in the analysis.

Enrichment fold change correlations were 96% to 99% across all species pairs (Figure S1). Of the 514 motifs that were enriched in at least one species, 73% (377/514) were commonly enriched across all five species (Fig. 1b), with a similar set of motifs enriched in accessible chromatin regions across species (Figure S2). One hundred and fourteen motifs were UMR-enriched for at least one species but not across all five. Some of these motifs showed highly variable lineage-specific patterns; several bHLH and SPL motifs were depleted in *H. vulgare* and *B. distachyon* yet enriched in *O. sativa* (Fig. 1c). While these variably enriched motifs are intriguing, we focused our subsequent analyses of motif gain and loss on the set of 377 commonly enriched motifs (Table S1) to trace how instances of evolutionarily stable motifs have turned over.

### Characterization of motif instances across 589 widely adapted species

**Figure 2:**
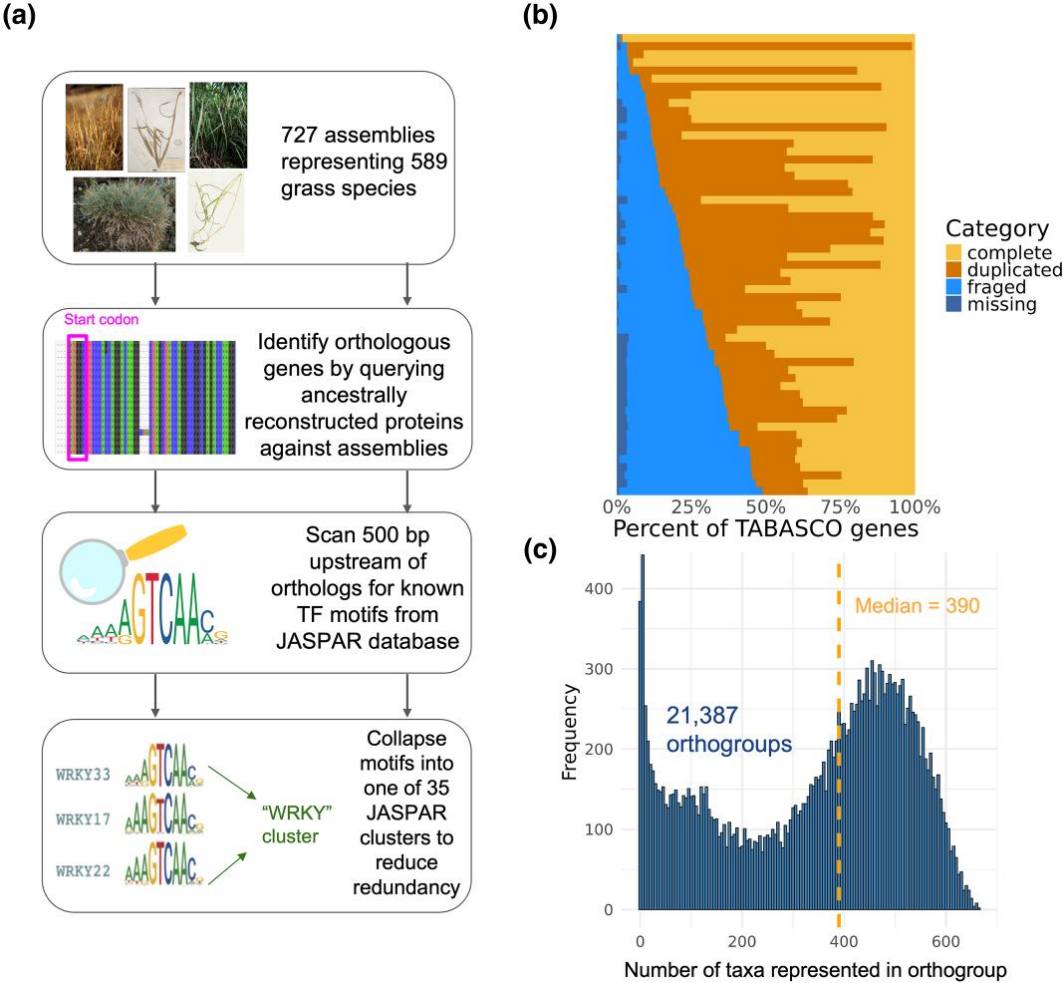
Summary of TF motif profiling across 727 assemblies. a) Depiction of bioinformatic pipeline to identify TF motif instances upstream of orthologous genes across taxa. Motif scanning was performed 500 bp upstream of the translation start sites of orthologous genes. b) Presence of single-copy grass orthologs (“TABASCO genes”) across 57 newly generated contig-level genome assemblies. The proportion of complete, duplicated, fragmented, and missing genes out of 5592 total TABASCO genes is shown. c) Distribution of taxa representation across 21,387 orthogroups in our dataset. The median number of taxa per orthogroup is plotted as a dashed vertical line.

To characterize cis-regulatory evolution on a large scale, we built a pipeline to quantify and compare motif occurrences across orthologous regions of hundreds of species (Fig. 2a). For this study, we used publicly available WGS short reads to generate genome assemblies for 57 species. While not highly contiguous, our WGS assemblies recovered a median of 4206 genes (75%) from a set of 5592 conserved grass genes in a BUSCO-like analysis (Fig. 2b; Table S2). In total, we amassed a dataset of 727 genome assemblies representing 589 diverse grass species using the 57 new WGS assemblies along with 211 public genome assemblies, 368 newly generated short-read assemblies from Schulz et al. unpublished data, 58 short-read assemblies from Schulz et al. (2023), and 33 highly contiguous assemblies from Stitzer et al. (2025) (Figure S4 and Table S2). Our dataset captures wide and repeated environmental adaptation across the grass family (Hsu et al., unpublished data).

To enable cross-species comparisons, we identified orthologous genes across taxa by querying a set of ancestrally reconstructed protein sequences against each assembly and retaining the primary alignment. We retained 21,387 orthogroups after filtering, each containing an average of 390 taxa (SD = 186) (Fig. 2c), indicating a high level of taxonomic representation suitable for robust comparative analyses. We scanned intervals 500 bp upstream of the aligned translation start sites for the set of 377 motifs with conserved UMR enrichment. While many important regulatory elements are present further upstream (or downstream) from genes, we considered only 500 bp upstream to maximize sample size given the limited contig lengths available in our short-read assemblies. We collapsed overlapping instances of similar motifs into a single merged interval to reduce redundancy for subsequent analyses, using 35 motif clusters delineated by JASPAR2024 (Table S1). The 35 motif cluster types differed widely in abundance and variability across taxa (Figure S5).

### Shared motif occupancy decays nonlinearly with increasing evolutionary divergence

To track the evolutionary gain and loss of motif instances in grasses, we quantified shared motif occupancy across species by comparing the number of motifs found at maize orthologs with those found at orthologs of the other 588 species (see Methods). Because many motif instances are not easily alignable, we chose to track the number of occurrences of each motif 500 bp upstream of the translation start site of each orthogroup, agnostic of position. This means that shared motifs may lack nucleotide-level conservation and that sharing can arise either from conservation or via independent gains at different sequence positions.

**Figure 3:**
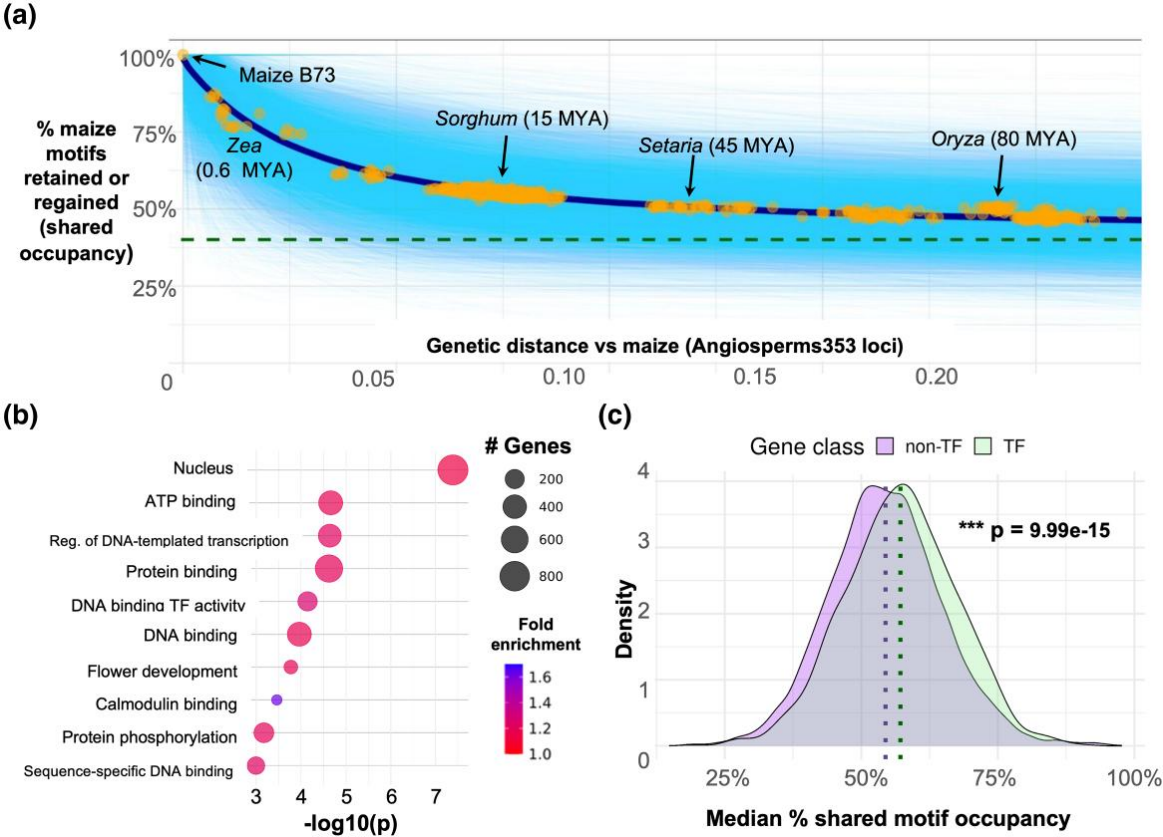
Shared motif occupancy decays nonlinearly and heterogeneously across gene classes. a) Percentage of maize motif instances retained or regained (shared occupancy) across 589 Poaceae species. Genetic distance was estimated using pairwise distances between maize and Poaceae species at the Angiosperms353 loci. Solid cyan lines represent exponential decay curves fit for 13,114 orthogroups. Points show the mean percentage of maize motifs with shared occupancy in each Poaceae species across all orthogroups, with an exponential decay curve depicted in a dark blue line. Dashed green line represents the mean percentage of motifs with shared occupancy across 100,000 random pairs of maize genes. Approximate divergence times from maize are shown for key taxa (Chen et al. 2022; Gallaher et al. 2022). b). Enriched GO terms for the orthogroups in the top quartile of shared motif occupancy. c) Shared motif occupancy at TF orthogroups (*n* = 1,190) versus at non-TF orthogroups (*n* = 11,482). Median values for each class are shown by dotted vertical lines. *P* value is from an asymptotic two-sample Kolmogorov–Smirnov test.

Shared motif occupancy decayed nonlinearly with increasing evolutionary divergence. Roughly 60% of maize motif instances were shared with *Sorghum* (∼15 million years diverged) at orthologous upstream regions, yet 50% were still shared with rice across ∼80 million years of evolution (Fig. 3a). On average, shared motif occupancy between orthologs did not decay to the level observed between random maize genes, approaching an asymptote of 48% shared occupancy (Fig. 3a). Decay curves were similar when defining shared motif occupancy relative to *Sorghum* (Figure S7a) and rice (Figure S7b) instead of maize. Additionally, motifs at maize orthologs that overlap a ChIP-seq peak for their cognate TF showed similar turnover levels to motifs without in vivo binding evidence (Figure S8). This suggests our motif turnover estimates are not substantially inflated by the many motifs lacking in vivo binding support. Shared occupancy of motif instances varied widely by gene, with most orthogroups approaching 40% to 60% shared occupancy between the most distantly related grasses (Fig. 3a). Coding sequence conservation explained minimal variance in shared motif occupancy at individual orthogroups (*R*-squared = 0.02) (Figure S9). A small number of highly conserved proteins, such as ribosomal proteins and proteins encoded in mitochondria and chloroplasts, exhibited very high rates of shared motif occupancy. However, the motifs detected near mitochondrial and chloroplast genes are likely artifacts of motif scanning, as these genes have distinct regulatory mechanisms compared to nuclear genes (Liere et al. 2011).

Orthogroups with higher shared occupancy were significantly enriched (Fisher's exact test, *P* < 0.05) for 126 Gene Ontology (GO) terms. Many of the strongest enrichments were related to regulatory function by TFs (Fig. 3b; Table S3). Specifically, TF genes (*n* = 1,190) exhibited slightly higher shared motif occupancy than non-TFs (*n* = 11,482) (TF mean = 57%, non-TF mean = 54%; Kolmogorov–Smirnov test, *P* = 9.99e−15) (Fig. 3c). The difference between TFs and non-TFs persisted when comparing shared motif occupancy between maize and sorghum at syntenic orthologs from Li et al. (2024) (Figure S10).

In addition to regulatory functions, we observed many significant GO terms among high shared occupancy genes related to signal transduction (e.g. “calmodulin binding”, “protein phosphorylation”, and “phosphorelay signal transduction system”), cytoskeleton (e.g. “actin filament organization”, “microtubule cytoskeleton organization”, and “cortical microtubule organization”), and development (“flower development”, “abaxial cell fate specification”, and “positive regulation of long-day photoperiodism, flowering”). Low shared occupancy genes were enriched for 114 terms including “ADP binding”, “defense response”, and “plant-type cell wall”. (Figure S11 and Table S3). GO terms that were not significantly enriched among either high or low shared occupancy genes included “response to abiotic stimulus”, “photosynthesis”, and “reproduction”.

Since many key regulatory elements are found further upstream than 500 bp from the translation initiation site, we re-ran our analyses using a 1 kbp upstream window and found a very similar motif decay pattern, the main difference being an elevated “baseline” level of shared motif occupancy due to the larger search space (Figure S12a). We also observed the same enrichment for TF activity among orthogroups with high shared occupancy (Figure S12b and c), indicating that our results are not very sensitive to window size, at least when considering gene-proximal regions.

### Environmentally associated motif gain/loss occurs at over a thousand genes

To investigate which motif instances are associated with environmental niche, we employed phylogenetic mixed models, which can be used to control for phylogenetic relatedness while associating genomic features with traits across species (Housworth et al. 2004). We characterized each species’ ecological niche as described in Hsu et al. (2025), summarizing into ten environmental principal coordinate (envPC) axes that together explain 75% of total ecological variability among the natural environments of grasses across the globe. The envPC1 primarily captures thermal variability across taxa, while envPC2 correlates with water availability (Figure S13).

**Figure 4:**
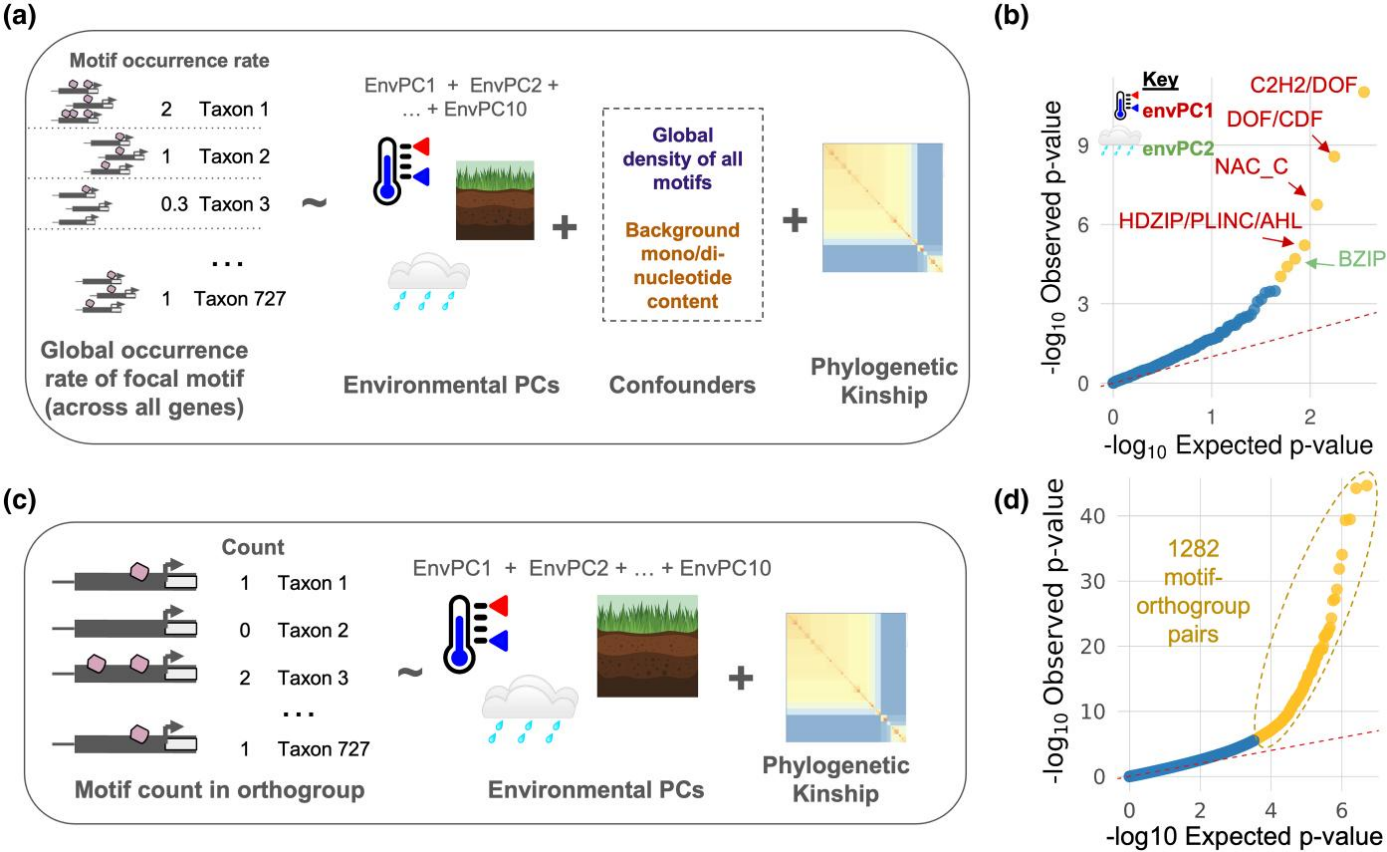
Turnover at over one thousand motif instances is associated with environmental niche. a) Setup of global occurrence association models. One phylogenetic mixed model was run for each of the 35 motif types. b) Quantile–quantile plot showing observed *P* values from the global occurrence models versus *P* values expected under the null hypothesis. Observed *P* values were calculated from Wald tests across 350 fixed effect envPC terms. Terms with FDR < 0.01 are shown in yellow. c) Setup of orthogroup-specific models. One model was run for each motif–orthogroup combination for a total of 540,015 models. d) Quantile–quantile plot for orthogroup-specific models. Observed *P* values were calculated for ∼5.1 million fixed effect envPC terms. Terms with FDR < 0.01 are shown in yellow.

We hypothesized that repeated gains or losses of particular motifs underlie environmental adaptation in grasses. We first tested whether global occurrence rates of motifs across taxa are associated with environmental niche, using the envPCs as proxies of environmental diversity (Fig. 4a). Since variable genome sizes have been hypothesized to alter the spacing of regulatory regions (Schmitz et al. 2021), we controlled for the overall density of all motifs as well as the background mono- and dinucleotide content in genomic background regions, which could influence the probability of detecting a motif by chance. Motifs with FDR < 0.01 were considered significant. Occurrence rates of C2H2-type zinc finger (C2H2/DOF), DNA binding with one finger (DOF/CDF), NAM, ATAF, and CUC (NAC), homodomain-leucine zipper/Plant Zinc Finger/AT-HOOK MOTIF NUCLEAR LOCALIZED (HDZIP/PLINC/AHL), cysteine-rich polycomb-like protein (CPP), and SCHLAFMUTZE (SMZ/RAP2.7/TOE2) motifs were significantly associated with envPC1 (Fig. 4b; Table S4). Additionally, the occurrence rate of basic leucine zipper (BZIP) motifs was significantly associated with envPC2. Each of these motifs was also significant (all *P* ≤ 0.002, not FDR-corrected) when using the permulation approach described by Saputra et al. (2021), demonstrating that our associations are not an artifact of phylogenetic structure. Using a 1 kbp upstream window for motif scanning produced largely similar results, with several additional significant associations uncovered (Figure S14 and Table S4).

We further hypothesized that repeated gains or losses of motif instances at particular orthogroups are associated with environmental adaptation. For each motif–orthogroup combination, we associated the number of motifs per assembly at an orthogroup with the ten environmental PCs after controlling for phylogenetic relatedness (Fig. 4c). Across 540,015 models, we scanned for signals of convergent motif gain or loss. Under an FDR < 0.01 significance threshold, 1282 unique motif–orthogroup combinations (1578 total) were significantly associated with at least one envPC (Fig. 4d; Table S5), suggesting convergent motif gain or loss across independent lineages. Of these top associations, 1562 (99%) were significant under permulation (*P* < 0.05, not FDR-corrected). The 16 associations that were not significant under permulation tended to have very high variance explained by phylogeny or other envPCs, leading to unstable estimates for the focal envPC. To more broadly compare our results with those derived from permulation, we also calculated *P* values using permulation for 100 random motif/orthogroup/envPC models. Our original *P* values very closely tracked those derived from permulation (Spearman rho = 0.98; Figure S15), indicating that our results do not show aberrant statistical behavior due to phylogenetic structure. Re-running the orthogroup-specific models with a 1 kbp upstream window yielded different top associations (Figure S14 and Table S5), indicating that this model setup is sensitive to the choice of window size.

### Gain of HSF/GARP motifs at an alpha-N-acetylglucosaminidase gene predicts cold adaptation

**Figure 5:**
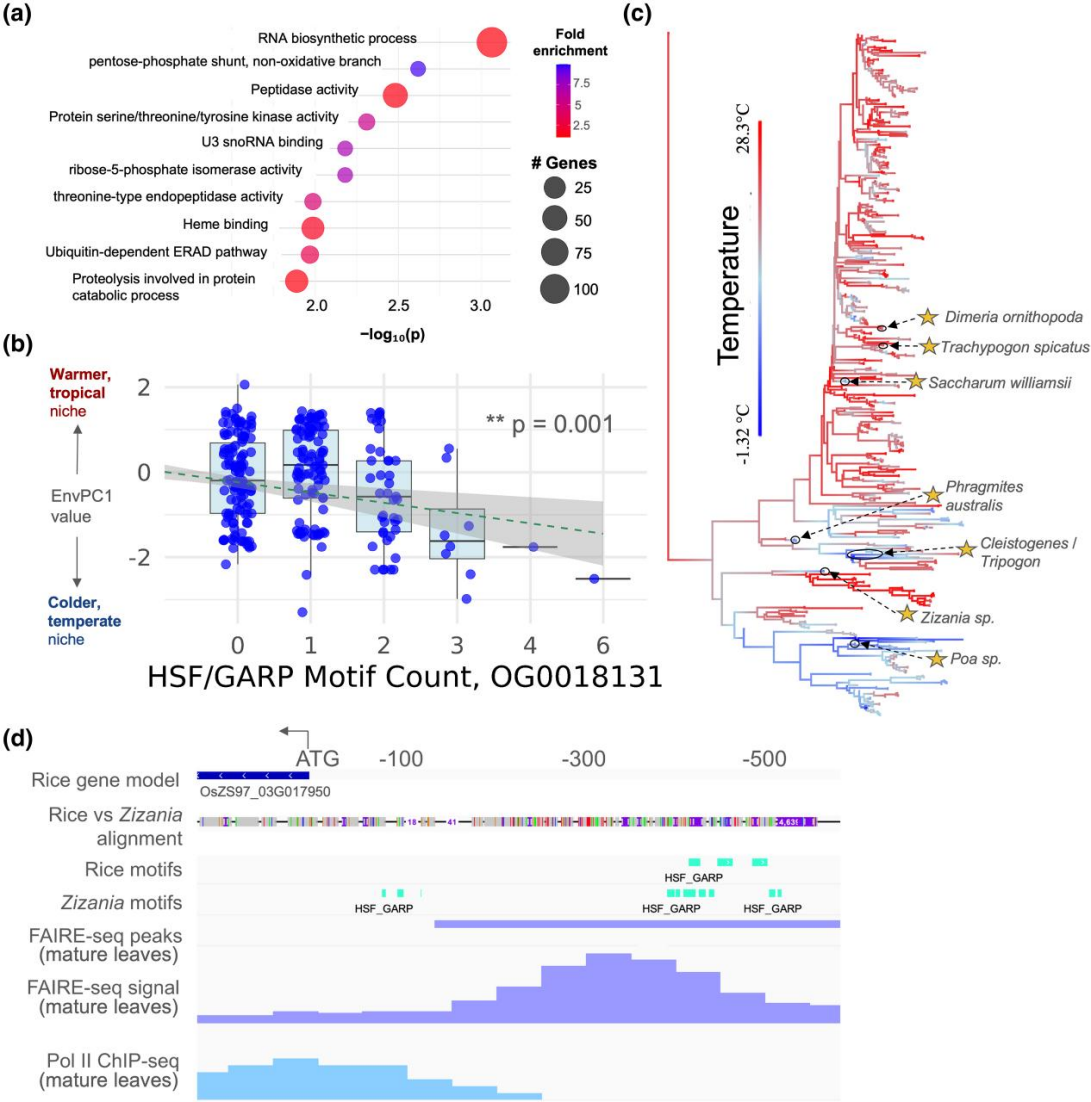
Exploration of top motif–orthogroup pairs. a) GO enrichment analysis for 1167 significantly associated unique orthogroups. The top ten most significant terms are shown. b) Environmental PC1 values for taxa with varying numbers of HSF/GARP motifs at OG0018131. Dashed line shows a linear model fit to envPC1∼HSF/GARP motif count. *P* value for the HSF/GARP model term is shown. c) Repeated gain and loss of HSF/GARP motifs at OG0018131 across the grass family. Taxa with three or more copies of HSF/GARP are starred. d) HSF/GARP motif variation between *Z. palustris* and *O. sativa* at an open chromatin region upstream of OG0018131 (OsZS97_03G017950). FAIRE-seq and RNA Polymerase II ChIP-seq data generated from mature leaves are shown.

We reasoned that the orthogroups most strongly associated with envPCs would be enriched for pathways and processes linked to abiotic stress responses. Indeed, a GO enrichment analysis of the 1167 significant unique orthogroups (the genes downstream of the regulatory regions scanned for motifs) identified 55 enriched terms (*P* < 0.05), many of which were related to known abiotic stress response processes such as oxidative stress response (“oxidoreductase activity”, “heme binding”) and degradation of misfolded proteins (“peptidase activity”, “ubiquitin-dependent ERAD pathway”, “proteolysis involved in protein catabolic process”, “ubiquitin-specific protease binding”, “endoplasmic reticulum unfolded protein response”, “threonine-type endopeptidase activity”) (Fig. 5a; Table S6).

We investigated some of the individual motif–orthogroup combinations related to these enriched processes. One of the motif–orthogroup combinations most strongly associated with envPC1 was a Heat Shock Factor/Golden2, ARR-B, Psr1 (HSF/GARP) motif at OG0018131 encoding an alpha-N-acetylglucosaminidase family protein. Alpha-N-acetylglucosaminidase is involved in N-glycan degradation (Ronceret et al. 2008), which has been connected to protein misfolding response under chilling in Arabidopsis (Ma et al. 2016) and alfalfa (Xu et al. 2024). Additionally, HSF TFs are strongly implicated in thermal stress responses (Guo et al. 2016), and in grasses, various HSFs have been linked to misfolded protein responses (Cheng et al. 2015) and chilling tolerance (Gao et al. 2024).

Increasing counts of HSF/GARP motifs at OG0018131 predicted lower envPC1 values (*P* < 0.001; Fig. 5b), with the association primarily driven by taxa containing three or more HSF/GARP instances. At least seven independent lineages had three or more copies of the motif (Fig. 5c), representing frost-tolerant genera including *Zizania* (wild rice), *Phragmites*, and *Poa*. Notably, only a fraction of independent cold adaptation events (very roughly 25%) were associated with expansions of this motif, indicating that the strength of convergence at this gene was relatively weak despite strong statistical support (Wald test, *P* = 1.89e−10). Rice contains multiple copies of the motif in an open chromatin region, and its cold-tolerant relative *Zizania palustris* contains additional copies (Fig. 5d).

A recent study documented allele-specific expression of the closest maize ortholog (Zm00001eb394460) under drought conditions (Engelhorn et al. 2025) with the gene also overlapping a MOA-seq bQTL, indicating variation in TF occupancy (Engelhorn et al. 2025). In rice, the gene is found within known QTLs for abiotic stress tolerance, including a 200 kbp GWAS peak (36 genes) that is associated with survival rate under chilling conditions (Khatab et al. 2022). Furthermore, by querying the EMBL expression atlas, we found that this gene and its homologs are differentially expressed in response to multiple abiotic stressors in diverse grasses (maize, sorghum, rice, *Brachypodium*, and barley) as well as poplar and soybean (Table S7).

## Discussion

Our results support a “stable motifs, variable binding sites” model of cis*-*regulatory evolution under which TF binding preferences remain conserved across deep divergence while individual TFBS across the genome are frequently gained and lost. The high overlap of UMR-enriched motifs across species suggests that minimal lineage-specific loss of binding preferences has occurred in grasses, consistent with the deep conservation of DNA binding domains observed within many TF families (Yamasaki et al. 2008; Weirauch and Hughes 2011; de Mendoza et al. 2013) and recent studies using DAP-seq to uncover conserved TF binding preferences across plant evolution (Baumgart et al. 2025; Zenker et al. 2025).

A few considerations should be kept in mind when interpreting these findings. First, the close similarity of motifs recognized by related TFs could lead to an overestimation of motif enrichment across multiple TFs if binding for a single TF is enriched. Additionally, our analysis does not rule out lineage-specific gains of binding site preference (perhaps via diversification of TF families), which could be investigated using *de novo* motif characterization. Quantitative estimates of binding affinity would be needed to more sensitively detect subtle shifts in binding preferences. Regardless, the high conservation of motif sequences we observed across distantly related species highlights that TFs in grasses share similar binding preferences, likely with transferable regulatory functions across species. Our implicit assumption is that UMR enrichment is evidence of motif function, although motif depletion can also be meaningful since selection on cis-regulatory regions may act to inhibit particular TF binding events (He et al. 2011). Additionally, many UMRs do not act as cis-regulatory elements, though we did observe similar enrichments in accessible chromatin regions for most motifs.

While much of grasses’ regulatory code appears to have been conserved across a hundred million years of evolution, our work demonstrates that extensive remodeling of individual cis-regulatory regions has occurred. Although some TFBS instances have been shown to be as strongly conserved as coding sequences (Heyndrickx et al. 2014), just 50% of the motif occurrences we observed were shared between maize and rice, which are roughly as genetically divergent as humans and mice (Gallaher et al. 2022). Since we quantified motif occurrences within a 500 bp window upstream of the translation start site, a “shared” motif across species does not imply nucleotide-level conservation, but rather that the motif occurrence is shared either by conservation or independent gains. Our collapsing of similar motifs could potentially underestimate TFBS turnover, since motifs counted as “shared” in our analysis may be divergent enough to be recognized by distinct TFs. On the other hand, TFBS present beyond 500 bp upstream of the translation start site might be misleadingly labeled as “lost” by our analysis. Overall, our estimates of shared occupancy are similar to those derived independently within Andropogoneae species by Stitzer et al. (2025) (∼60% shared between maize and sorghum) and from ChIP-seq in maize and rice (20% to 50% shared GLK binding events in proximal promoter regions). Estimates relying on nucleotide-level alignment between placental mammals of comparable divergence are even lower (10% to 22% shared) (Schmidt et al. 2010).

Despite extensive turnover overall, some motif instances appear to persist over deep evolutionary time scales. Shared motif occupancy at orthologous regions decays nonlinearly and tends to approach a minimum level of shared occupancy between 40% and 60% for most grass orthogroups. This suggests that regulatory regions may often contain a handful of strongly conserved motif instances alongside many motifs that are rapidly turned over. Our finding that regulatory genes, and specifically TFs, have slightly higher shared motif occupancy suggests that a large amount of cis-regulatory evolution within regulatory networks occurs at terminal target genes rather than just at a handful of key regulators. This finding supports a model in which TFs and other highly pleiotropic genes exhibit greater constraint, in line with past theoretical and empirical work (Prud’homme et al. 2007; Chesmore et al. 2016; Nocchi et al. 2024), but see Khaipho-Burch et al. (2023). Future studies could explicitly test whether the degree of pleiotropy of a gene predicts conservation of its TFBS. Alternatively, background selection on the target gene could be driving conservation of proximal TFBS rather than selection on the TFBS itself, though the low correspondence between coding sequence and shared motif occupancy suggests that this effect is minimal.

Our environmental association models suggest that cis-regulatory changes in grasses are not concentrated at a handful of key loci, but occur pervasively across a wide set of diversely functioning genes. We observed weakly convergent motif gain and loss at over a thousand genes with diverse molecular functions, underscoring that environmental adaptation involves altering regulation of a highly diverse set of genes, not just modifying a handful of pathways or processes. These results are consistent with theoretical findings that suggest that global adaptation of complex traits tends to be associated with many small-effect regulatory changes (Prud’homme et al. 2007). This challenges attempts to leverage cross-species insights for crop genetic engineering. Still, characterization and editing of key TFBS remains a promising strategy for precisely tuning expression levels.

Our results suggest that phylogenetic mixed models may be an effective approach to nominate key candidate loci using large-scale comparative genomics. With more sequenced genomes available, the ability of such comparative genomic approaches to detect convergently evolving genome features will improve (Smith et al. 2020), particularly for loci of small effect. Future studies could build on this framework by pairing large-scale comparative genomics screens with detailed molecular characterization of the top candidate loci. For example, a variety of mechanisms could underlie the HSF/GARP motif variation we observed at OG0018131. Gains of HSF/GARP motifs at this locus could alter TF binding affinity of HSF/GARP TFs to the region, or perhaps permit combinatorial binding of multiple distinct HSF/GARP TFs that recognize similar motifs. Further work could validate the functional effect of this motif and its relationship with environmental adaptation.

A number of trade-offs were required to scale up our analyses across hundreds of complex plant genomes. The relatively high proportion of gene-proximal motifs intersecting open chromatin and TF binding regions suggests that many gene-proximal motifs are likely to be bound by TFs in vivo. Still, a significant fraction of the motif instances we characterized are likely to be nonfunctional. Additionally, studies have found that only a fraction of TF binding changes are due to motif variants (Reddy et al. 2012; Krieger et al. 2022), underscoring that TF binding assays, though far less scalable, currently offer more reliable estimates of TFBS than sequence-based prediction. Deep learning models hold promise going forward for scalable characterization of cis-regulatory regions. The ability of such models to represent the orientation, positioning, and co-binding context of cis-regulatory elements can offer a more nuanced picture of cis-regulatory evolution beyond simple gain and loss of motifs.

Our study focused on characterizing motif variants in proximal upstream regions due to limited assembly contiguity. However, changes to distal cis-regulatory elements (Clark et al. 2006) and trans-acting factors also contribute strongly to regulatory adaptation and were not considered in our analyses of motif gain and loss. Another limitation of our approach is that many duplicated gene copies, particularly in the numerous polyploid taxa represented in the dataset, were filtered out. Additionally, because we filtered out small orthogroups with fewer than 200 taxa represented in our orthogroup-specific association models, lineage-specific genes that may be key to adaptation were not considered. Similarly, the reliance on convergent evolution in our association models may miss adaptive mechanisms that are influenced by evolutionary contingencies and constraints; e.g. lineages using C4 photosynthesis may have different physiological strategies “available” to them than C3 lineages. Future comparative genomic analyses will benefit from complementing broad phylogenetic comparisons with comparisons of closely related species that largely share the same physiology and genetics. Moreover, integrating more detailed estimates of ecological context into population genetic analyses of key taxa would offer greater resolution and nuance beyond our broad species-level estimates of adaptation.

## Conclusion

We performed large-scale comparative genomic analyses across 589 grass species to investigate the cis-regulatory basis of diversification and environmental adaptation in grasses. We found that grasses share a deeply conserved regulatory code, with 377 cis*-*regulatory sequence motifs conserved across diverse species. We documented widespread gain and loss of specific motif instances across the grass family, suggesting extensive reorganization of orthologous cis-regulatory regions. Cis-regulatory changes do not appear to be highly concentrated at particular classes of genes, though they occur slightly less frequently at regulatory genes such as TFs. One thousand and two hundred eighty-two motif–orthogroup combinations show evidence of convergent gain and loss of motifs associated with environmental variables. However, even the most strongly significant motifs are only weakly repeatable, underscoring the diversity of cis-regulatory routes to environmental adaptation.

## Materials and methods

### TF motif scanning

We downloaded 805 TF motifs and clusters from the 2024 JASPAR CORE non-redundant plant collection (Rauluseviciute et al. 2024). This set of motifs has strong experimental evidence from TF binding assays such as DAP-seq and ChIP-seq primarily performed in *Arabidopsis*. All motif scans were performed using FIMO from the MEME suite v5.5.7 (Grant et al. 2011) with a *P* value threshold of 0.0001 and the parameters --max-strand --no-qvalue --skip-matched-sequence --max-stored-scores 100000000. Homogenous background mono- and dinucleotide frequencies (=0.25 per nucleotide) were used to avoid species-specific biases in motif detection thresholds. Seven hundred four of the 805 motifs in the JASPAR collection were detectable using our parameters, with the rest containing insufficient information content to enable statistically significant matches with FIMO. While we experimented with using quantitative scores based on the strength of the FIMO match to represent motif occupancy rather than simple presence/absence, we found that this did not increase biological signal in our analyses so we proceeded with presence/absence counts. A visual example of our motif scanning results upstream of the *ZmICE1* gene can be seen in Figure S6.

### Motif enrichment analysis

We downloaded processed UMRs for five diverse grass species (*S. bicolor*, *O. sativa*, *B. distachyon*, *Z. mays*, *H. vulgare*) (Crisp et al. 2020). Background sequences were generated by dinucleotide shuffling each UMR region 100 times, preserving local sequence composition. Motif scanning was performed using FIMO on both UMR and background sequences. The fitdistrplus::fitdist function (Delignette-Muller and Dutang 2015) was used to fit probability distributions to background motif counts. Two-tailed *P* values were then calculated to identify significantly over- and under-represented motifs in UMR regions. We quantified how many motifs were significantly over-represented (FDR < 0.01, Fisher's exact test) within and between species. The 377 commonly enriched motifs were used for subsequent analyses.

For accessible chromatin enrichment, we downloaded bulk accessible chromatin regions for *S. bicolor*, *O. sativa*, *B. distachyon*, *Z. mays*, *H. vulgare*, *Setaria viridis*, and *Arabidopsis thaliana* as processed by Lu et al. (2019) and performed enrichments as described above. We also compared motif enrichments in maize using Andropogoneae conserved noncoding regions (Stitzer et al. 2025) with intronic regions filtered out, processed scATAC peaks merged across all cell types (Marand et al. 2021) that we uplifted to B73 v5 coordinates, and processed MOA-seq peaks from 25 maize hybrids that had been mapped onto B73 v5 coordinates and merged (Engelhorn et al. 2025). For the maize comparison, we compared enrichments using two strategies to generate background regions: (i) the dinucleotide shuffling approach described above and (ii) permuted genomic regions that we generated using bedtools shuffle v2.31.1 (Quinlan and Hall 2010) with default parameters. We found from this analysis that dinucleotide shuffling yields much more enrichment consistency across feature types compared to using permuted genomic regions as background (Figure S3). The high enrichment variability across feature types with the permuted background is likely due to variable dinucleotide content across feature types which influences the odds of a motif match occurring by chance alone.

### Compiling genomic dataset

We compiled a dataset of 727 genome assemblies representing 589 diverse grass species by combining 217 publicly available assemblies from NCBI (Sayers et al. 2024), Phytozome (Goodstein et al. 2012), and CoGE (Lyons and Freeling 2008) with 33 long-read assemblies from Stitzer et al. (2025) and 550 additional assemblies from short reads using the high-throughput assembly pipeline described in Schulz et al. (2023) (Table S2). For this study, we assembled 57 new short-read assemblies from WGS raw reads deposited in the NCBI SRA database (Sayers et al. 2024). We downloaded WGS data from all Poaceae species lacking an existing genome assembly if at least 15 GB of WGS data was available for that species. We then assembled the SRA genomes de novo using Megahit v1.2.9 (Li et al. 2015) with minimum *k*-mer size = 31 and default parameters for other assemblies, as described in Schulz et al. (2023). If >30 GB WGS data was available for the largest WGS accession for a species, we generated a single assembly using the largest accession. If <30 GB data was available for the largest accession, we merged WGS data from multiple accessions to improve assembly completeness. We performed three QC steps on our SRA assemblies. First, we ran Kraken2 v2.1.3 (Lu et al. 2022) on raw reads (subsampled to a depth of 10M reads) using the PlusPFP database to verify sample identity on a rough taxonomic scale and flag accessions with high levels of bacterial or fungal contamination. Second, we visualized the assemblies on a *matK* phylogenetic tree against labeled *matK* sequences from the BOLD database (Ratnasingham et al. 2024) to rule out obviously mislabeled accessions. To generate *matK* alignments, we downloaded a complete *matK* CDS sequence for *Streptochaeta angustifolia* (GenBank: AF164382.1) (Hilu et al. 1999). We queried the *S. angustifolia* sequence against all WGS short-read assemblies using minimap2 v2.17 (Li 2018) with the parameters -ax asm20 –eqx -I 100g –secondary=no, extracting the primary alignment in each assembly. Using the extracted *matK* sequences from our WGS assemblies and 7,338 Poaceae *matK* sequences from the BOLD database, we performed multiple alignment with mafft v7.520 (Katoh et al. 2002) --auto. Then we constructed a phylogenetic tree using raxml v8.2.13 (Stamatakis 2014) with the GTRGAMMA model. We calculated assembly contiguity statistics using assembly-stats v1.0 (Challis 2017). As a final assessment of assembly quality, we quantified assembly completeness using TABASCO, a BUSCO-like assembly metric designed specifically for use with grasses (Schulz et al. 2023) which labels a set of 5592 query genes as “complete”, “duplicated”, “fragmented”, or “missing”.

### Orthogroup construction

Across the 727 genome assemblies, we selected 32 representative high quality long-read assemblies to construct orthogroups. In order to avoid potential annotation biases, we ran Helixer (Stiehler et al. 2021) to annotate each of the representative genomes and extracted the protein sequences. Based on protein sequence homology, orthogroups were constructed using OrthoFinder v2.6.4 (Emms and Kelly 2019). In total, we obtained 22,503 orthogroups with homologous sequences represented by more than eight of the representative genomes. From the multiple sequence alignment of each orthogroup, we reconstructed the ancestral protein sequence using the R/phangorn package (Schliep 2011). We used ancestral sequences of the orthogroups to query the orthologs in all 727 genomes with miniProt v0.13.0 (Li 2023).

### Phylogenetic characterization

We calculated phylogenetic relatedness among the 727 studied genomes using the Angiosperms353 loci (McDonnell et al. 2021). We identified the orthogroups homologous to the Angiosperms353 loci using miniProt (v0.13.0) and generated gene trees for each of the orthogroups using RAxML v8.2.12 (GAMMA + GTR). ASTRAL-Pro v2 (Zhang and Mirarab 2022) was used to reconcile the species tree based on the gene trees. We generated a matrix of shared branch length among any pair of tips in the species tree to represent the relatedness between them. This matrix (hereafter phyloK matrix) was included in our phylogenetic mixed models to account for shared macroevolutionary history among species. Using the concatenated alignment of the Angiosperms353 loci, we calculated pairwise genetic distance among the 727 taxa based on the K81 model implemented in R/ape::dist.dna() function. We obtained divergence time estimates for *Zea-Tripsacum* from Chen et al. (2022) and estimated divergence time across Poaceae from Gallaher et al. (2022).

### Motif annotation in orthologous upstream regions

Alignments were filtered to retain primary alignments that did not contain a frameshift or a premature stop codon, and that began with a start codon. To approximate 5′ UTR and promoter regions across assemblies, 500 bp sequence was extracted upstream of the aligned start codon. 500 bp was chosen as a conservative estimate of 5′ UTR and promoter regions to maximize species representation in the dataset given the limited contig lengths for the short-read assemblies. If 500 bp upstream sequence was not available, or if the sequence contained >5% Ns, the sequence was discarded from analysis. To minimize redundant, overlapping motif annotations, we collapsed similar overlapping motifs into a single interval. We defined motif similarity based on membership within the same matrix cluster, as designated by JASPAR 2024 (Rauluseviciute et al. 2024) (Table S1). The 377 motifs with conserved UMR enrichment represented 35 unique clusters. We manually annotated each of the 35 clusters with a descriptor of the motifs contained within (e.g. “HSF/GARP” if the cluster contained motifs from HSF and GARP TFs.) After collapsing overlapping motif instances by cluster, we quantified the number of cluster instances per upstream region for each assembly.

### Motif abundance and variability

We used our motif counts in 500 bp upstream regions to estimate (i) the median occurrence rate and (ii) coefficient of variation of each motif cluster across assemblies. We calculated occurrence rates of each motif for each assembly by dividing the total number of instances of a particular motif in 500 bp upstream regions by the number of 500 bp upstream regions represented in that assembly, effectively calculating the average number of motif instances per upstream region. We then calculated the median and coefficient of variation for motif occurrence rates across all assemblies.

### Shared occupancy of motif instances

Shared motif occupancy was defined for each assembly relative to maize B73, with the percent shared occupancy of maize motifs at a single orthogroup defined as (# motifs present at the target assembly gene/# of motifs present at the maize ortholog). As with most estimates of TFBS turnover, we did not consider motifs present in other lineages but absent in maize. For example, shared occupancy at a single ortholog with three motif types present might be quantified as follows:

**Table:** 

	Motif A instances	Motif B instances	Motif C instances	Total maize motifs present
Target assembly	1	0	2	2
Maize	1	1	1	3
% shared occupancy	100%	0%	100%	67%

We calculated shared motif occupancy relative to maize both at individual orthogroups (“local”) and across all 13,114 orthogroups present in maize (“global”). For local and global shared occupancy, we plotted shared motif occupancy against genetic distance (calculated using the Angiosperms353 loci as described above). We fit exponential decay curves of the form


y=a×exp(−bx)+c

to the relationship between genetic distance (x) and shared motif occupancy (y).

To identify functional enrichments for orthogroups with high and low shared motif occupancy, we calculated the mean percent shared occupancy across taxa for maize motifs. To minimize biases from lineage-specific genes when calculating mean shared occupancy, we only considered orthogroups containing at least 200 taxa. Using the mean shared occupancy values for each orthogroup, we identified orthogroups within the top and bottom quartiles of shared motif occupancy. The top and bottom quartiles each corresponded to 3,168 maize genes, which we mapped to orthogroups using miniProt (v0.13.0) between the maize sequences and the reconstructed ancestral sequences of the orthogroups in this study. We then performed GO enrichment using the R/topGO package (Alexa and Rahnenführer 2009) with the “weight01” algorithm, which considers hierarchical relationships among terms. For background genes, we used the remaining 10,822 orthogroups not contained in the target quartile. Using maize B73 v5 GO annotations downloaded from MaizeGDB (Woodhouse et al. 2021), we tested for significant cellular component, biological process, and molecular function terms and calculated *P* values from Fisher statistics. Due to the topology-aware approach that topGO's “weight01” algorithm uses, the *P* values returned are considered to be already corrected for multiple testing. Therefore, we chose to present raw *P* values for the top GO terms rather than FDR-corrected values.

To compare shared motif occupancy at TF versus non-TF genes, we downloaded a list of maize TFs from Grassius (Gavgani et al. 2023) corresponding to 1,190 distinct orthogroups. Using orthogroup-level measures of shared motif occupancy, we used an asymptotic two-sample Kolmogorov–Smirnov test to evaluate the difference in shared occupancy between TFs and non-TFs (all orthogroups not contained in the Grassius list).

We wanted to determine whether false-positive motif hits without evidence of in vivo binding were causing us to overestimate motif turnover rates. To quantify this, we compared shared motif occupancy at genes with ChIP-seq support versus all genes. We downloaded processed ChIP-seq data from MaizeGDB for four maize TFs (Tu et al. 2020) matched to motifs used in our analyses: ereb17 (MA1818.1), glk53 (MA1830.2), bhlh47 (MA1834.2), and hb34 (MA1824.2). For each TF, we identified a set of orthogroups for which the 500 bp upstream region of the maize ortholog intersected with a ChIP-seq peak. Then, for the motif clusters corresponding to each ChIP-seq TF, we quantified shared occupancy motif instances between maize and every assembly across (i) all orthogroups and (ii) all orthogroups intersecting a maize ChIP-seq peak.

### Environmental characterization

We followed an environmental characterization pipeline previously described by Hsu et al. (2025) to characterize the environmental niches of the studied species. Briefly, for each species, we retrieved the geographical coordinates of occurrence from BIEN (Maitner et al. 2017) and GBIF.org (2025) GBIF Occurrence Download (Derived dataset GBIF.org) and obtained various environmental features for each occurrence (Hsu 2025). We were able to characterize the habitat environments for 706 out of 727 taxa and thus excluded taxa where we were unable to curate environmental data from subsequent analysis (Hsu et al., unpublished data). The variation in habitat environments among species was summarized into environmental principal components (“envPCs”). Each envPC captures a different combination of spatial and climatic patterns (Figure S13), with the multivariate decomposition process resulting in synthetic variables (the envPCs) that preserve important statistical properties, such as orthogonality, lack of autocorrelation, and normality. The top 10 environmental PCs were modeled as predictor variables in our phylogenetic mixed models.

### Cross-species environmental association models

For models associating global motif occurrence rates with environment, we fit 35 phylogenetic mixed models of the following form with ASReml-R v4.2 (Butler et al. 2009), with one model per motif and one observation per assembly in each model:


$$\begin{array}{ll} Focal\;motif\;occurrence\;rate & ∼environmental\;PC1\\ & +\;environmental\;PC2+\;\ldots \\ & +environmental\;PC10\\ & +\;Global\;motif\;density(all\;motifs)\\ & +\;Mono/di\\ & -nucleotide\;frequency\;PCs\;1\text{–}5\\ & +\;phyloK \end{array}$$

The phylogenetic relationship matrix (phyloK) was fit as a random effect to control for shared evolution across species. All other predictor variables were fit as fixed effects. Occurrence rates for each focal motif were calculated as described in the “Motif abundance and variability” section. We quantified global motif density (across all motifs) for each assembly by summing all 35 individual motif occurrence rates. Mono/dinucleotide principle coordinates were used as covariates to control for background genome characteristics such as GC content, which influence motif detection rates. To estimate mononucleotide and dinucleotide frequencies in background genomic regions, we extracted sequences for all introns shorter than 150 bp using the miniProt alignments previously described in the “Orthogroup construction” section. We used the fasta-get-markov function from the MEME suite with -m 1 to calculate mono/dinucleotide frequencies, and five principle coordinate axes were generated from these frequencies using the R/ade4 package (Dray and Dufour 2007). We fit the envPC terms as fixed effects, along with the mono/dinucleotide content and global motif density covariate terms. We calculated *P* values for each envPC term using Wald tests. We plotted the distribution of the 350 observed *P* values against *P* values expected under a uniform distribution to identify deviations from the null hypothesis.

For orthogroup-specific models of motif-environment association, we used ASReml-R v4.2 to run phylogenetic mixed models of the following form, with one observation per assembly:


$$\begin{array}{ll} Motif\;instances\;in\;orthogroup & ∼environmental\;PC1\\ & +\;environmental\;PC2+\;\ldots \\ & \;+\;environmental\;PC10\\ & +\;phyloK \end{array}$$

Environmental PCs (envPCs) were specified as fixed effects with the phylogenetic relationship matrix (phyloK) as a random effect to control for shared evolution across species. In total 540,015 models were run (# of motifs × # of orthogroups). To minimize model instability, we filtered out motifs occurring at an orthogroup in fewer than ten taxa and required orthogroups to contain at least 200 taxa. Wald tests were performed on each envPC term to calculate *P* values. We plotted the distribution of the ∼5 million observed *P* values against *P* values expected under a uniform distribution to identify deviations from the null hypothesis (no association between environment and motif occurrence). To visualize how motif gain/loss is associated with temperature adaptation events, we reconstructed ancestral temperature values across the Poaceae phylogeny using the contMap function from phytools v2.3-0 (Revell 2024).

We used phylogenetic permulations (Saputra et al. 2021) to check the robustness of our association model results. It was computationally intractable to run permulations across all 5 million associations, so we permulated (i) 100 random motif/orthogroup/envPC models and (ii) all of our top association model hits from the global and orthogroup-specific models. For each model, we permulated the focal envPC trait 1000 times while holding the other envPCs constant and ran association models to generate null distributions of Wald statistics for comparison with the empirical statistics.

### Visual comparison of orthologous regions

To compare orthologous upstream regions, we performed pairwise whole-genome alignments between *O. sativa* var. ZhenShan97 and *Zizania palustris* using Anchorwave v.1.2.2 (Song et al. 2022) with parameters -R 2 -Q 1. We generated chain files from Anchorwave alignments using a custom “MAFtoChain” script, then lifted over motifs into rice coordinates using CrossMap v0.7.3 (Zhao et al. 2014). To visualize open chromatin regions, we used RiceENCODE to download processed FAIRE-seq and RNA Polymerase II ChIP-seq tracks, both sampled from mature leaves from Zhao et al. (2020).

## Supplementary Material

msaf324_Supplementary_Data

## Data Availability

The short-read genome assemblies generated for this study are available at https://figshare.com/s/bc19e8f5dae558834cc2. Code to generate the analyses and figures can be found at https://github.com/maize-genetics/poaceae_tfbs.
