# Supplementary material for: Widespread turnover of a conserved cis-regulatory code across 589 grass species
Source: Mol Biol Evol. 2025 Dec 10;43(1):msaf324. doi: 10.1093/molbev/msaf324 (PMC12819352; doi:10.1093/molbev/msaf324)
Supplement: msaf324_Supplementary_Data [file msaf324_supplementary_data.zip › Hale_Supplementary_Appendix_2025.08.23 .pdf]

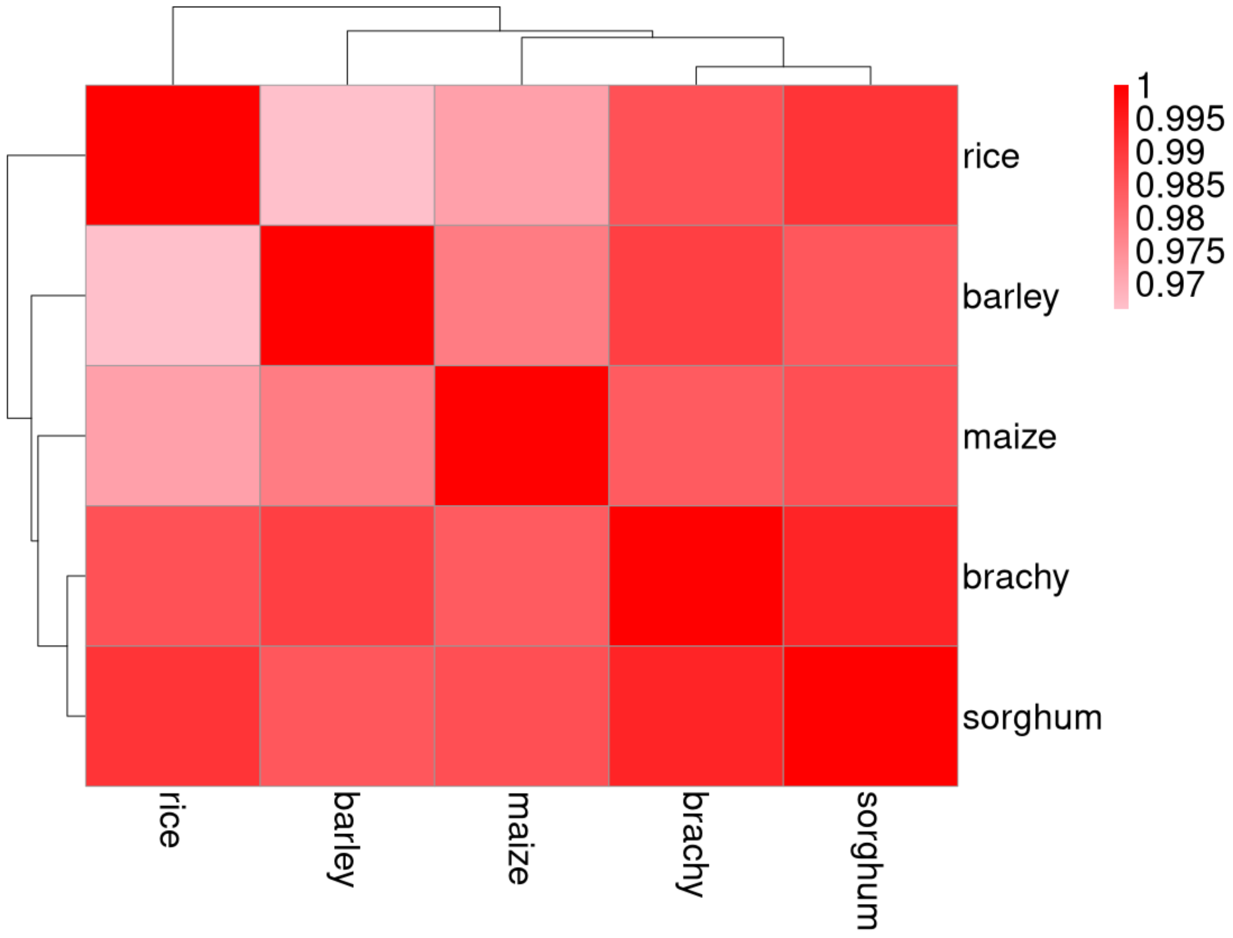

Supplementary Figure 1. Fold-change UMR enrichment correlations across species. Pearson correlations across 704 motifs are shown.

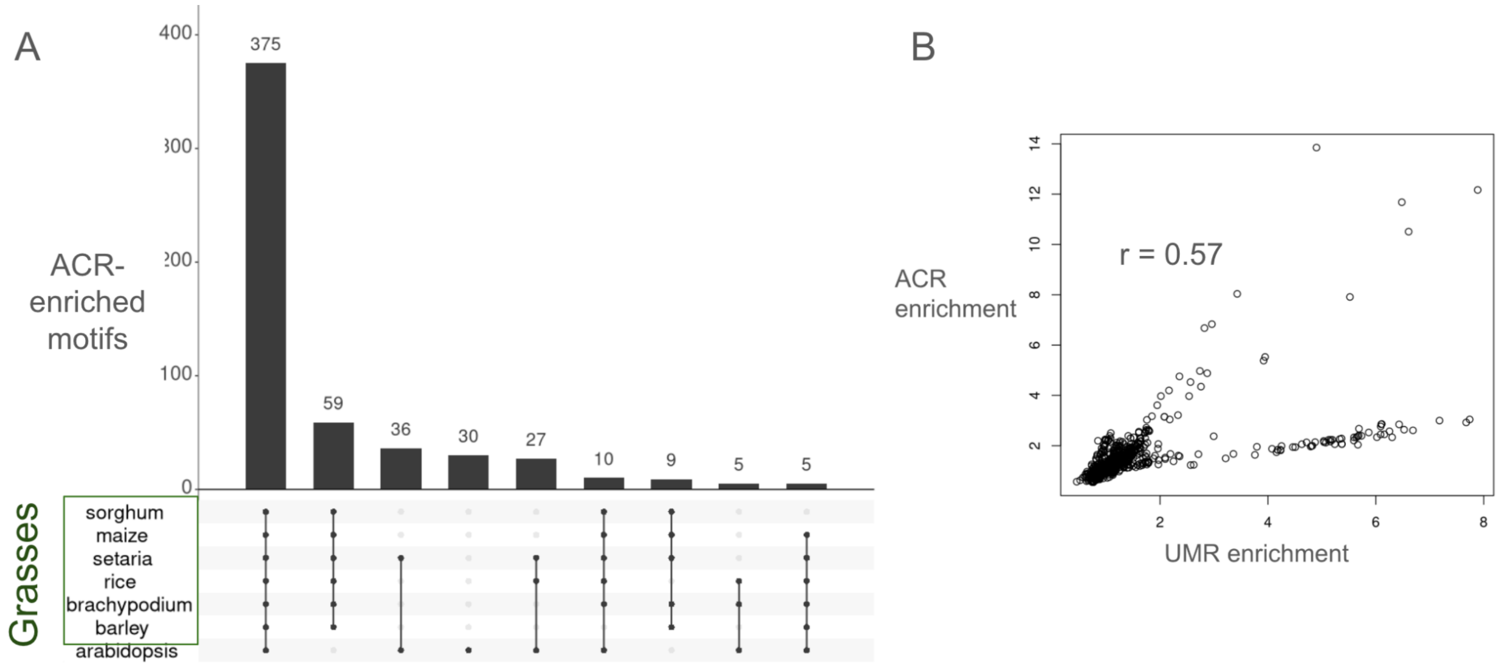

Supplementary Figure 2. Motif enrichment in accessible chromatin regions.

a) Enrichment of transcription factor motifs in accessible chromatin regions (ACRs) across species. The intersection bars show the number of enriched motifs for each species set. Intersections with fewer than five motifs are not shown. 336 / 377 (89%) of the shared UMR-enriched motifs were also commonly enriched in ACRs of grass species.

b) Pearson correlation between UMR enrichment fold-change and ACR enrichment fold change across motifs in maize

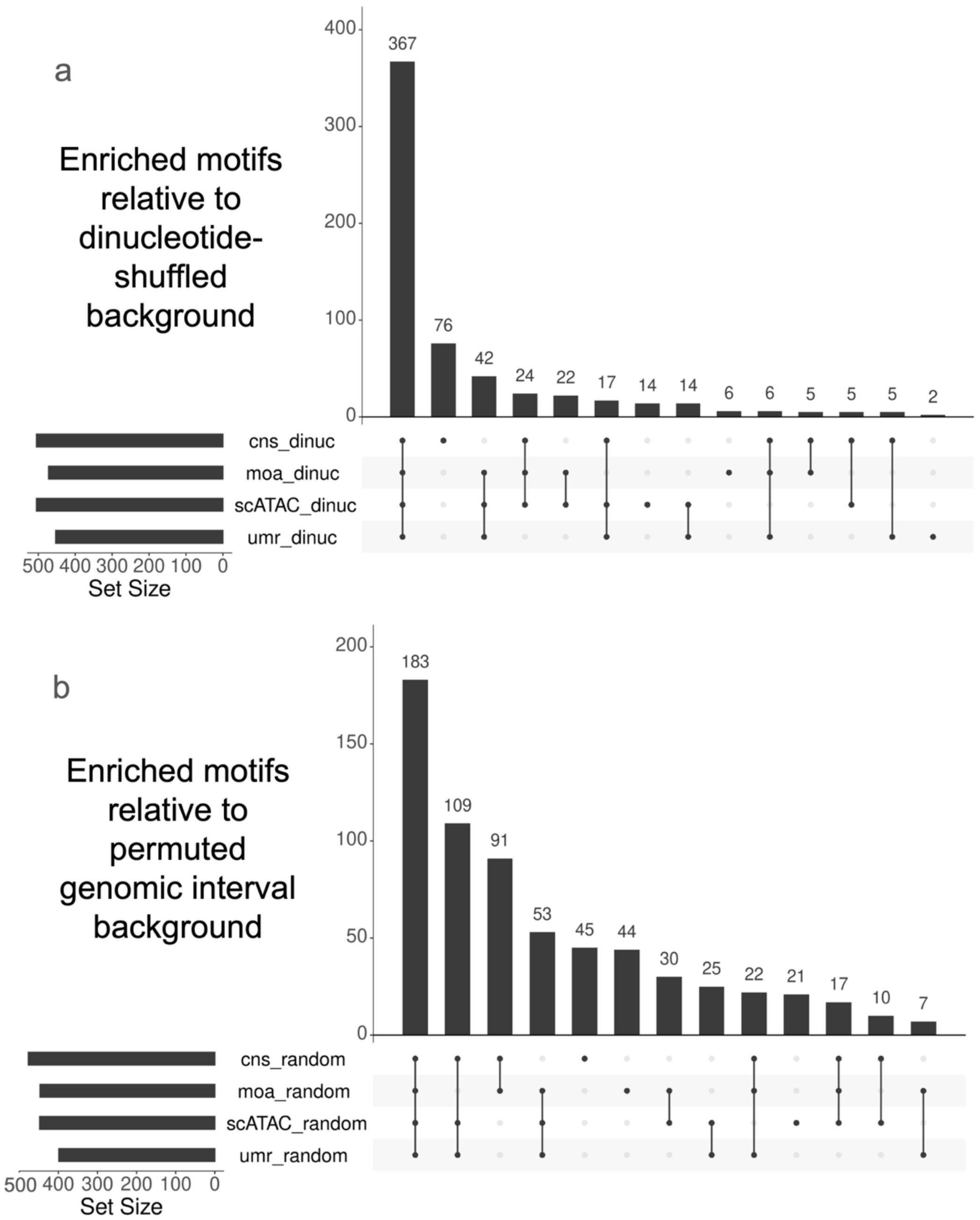

Supplementary Figure 3. Motif enrichment across maize feature types and background interval types.

a) Shared enrichment of motifs across conserved non-coding sequences, MOA-seq peaks, merged scATAC peaks across all cell types, and unmethylated regions. Enrichments were calculated relative to background regions obtained via dinucleotide shuffling of empirical regions.
b) Shared motif enrichment across feature types, with permuted genomic intervals (shuffling locations across the genome) used as background.

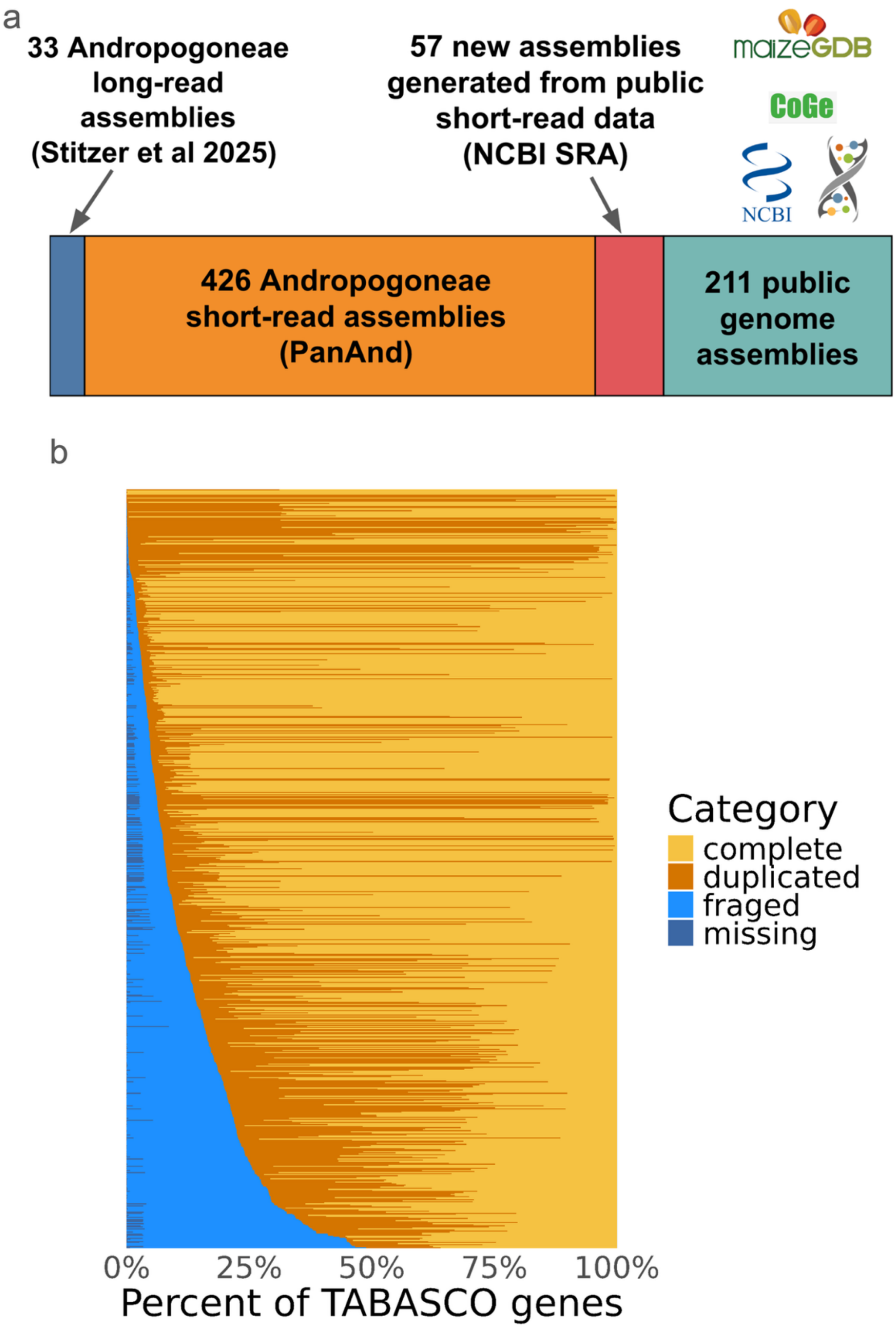

Supplementary Figure 4. Breakdown of genome assemblies used in this study.

a) Origins of 727 genome assemblies, representing 589 distinct species, that were used for analyses.
b) TABASCO scores representing assembly completeness across all 727 assemblies used in the analysis.

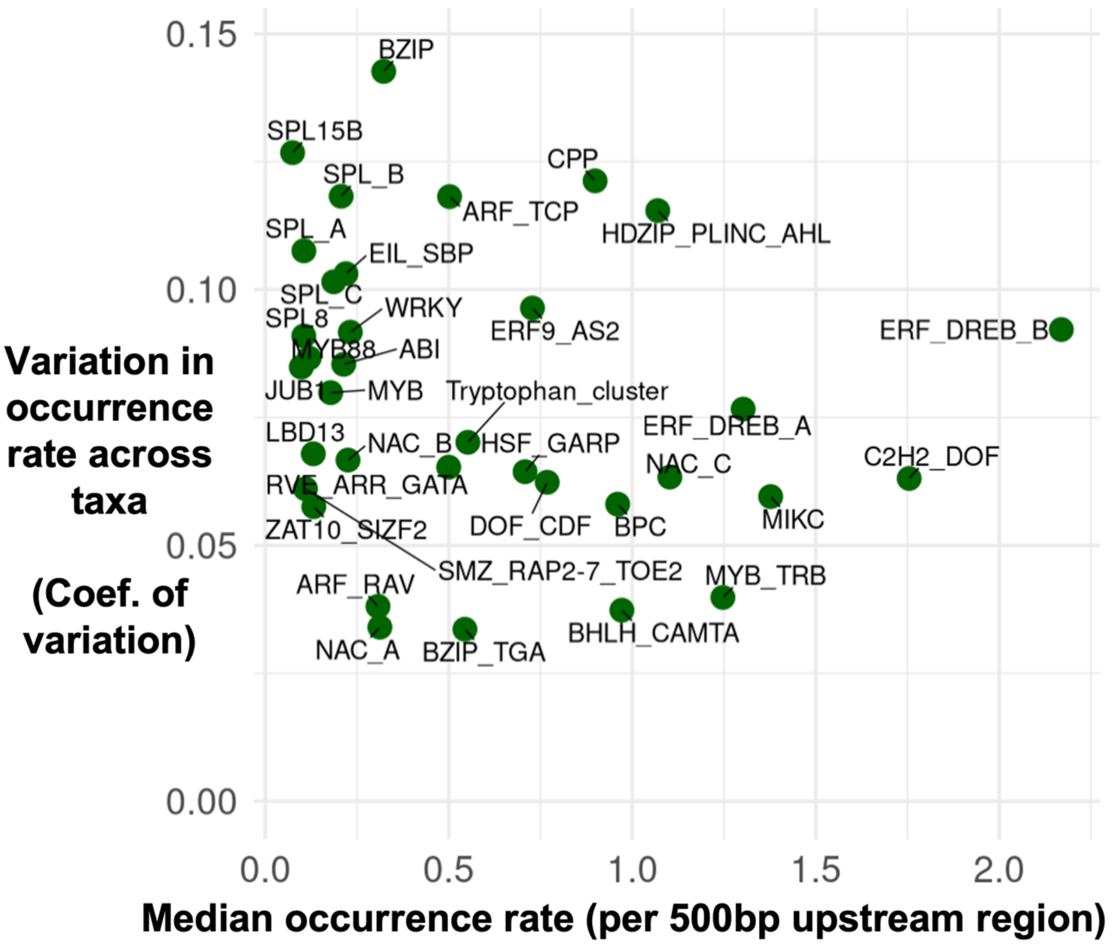

Supplementary Figure 5. Abundance and variability of motif cluster types.
Mean occurrence rates were calculated across all promoter regions in each assembly. The median and coefficient of variation across assemblies are plotted.

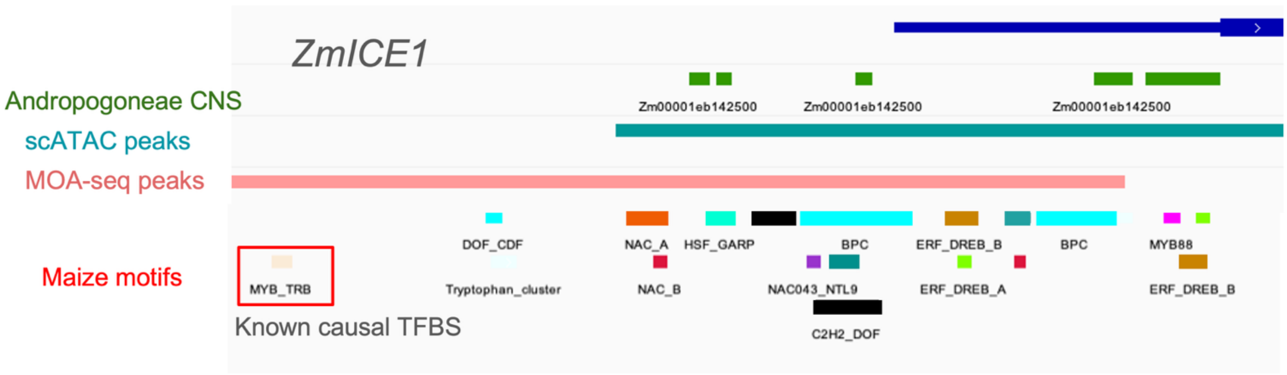

Supplementary Figure 6. Example motif annotation upstream of *ZmICE1*.
A known causal TFBS variant from (Jiang et al. 2022) is highlighted in the red box. Andropogoneae conserved non-coding sequences (CNS) are from (Stitzer et al. 2025). scATAC peaks are merged across all cell types from (Marand et al. 2021). MOA-seq peaks were merged across maize hybrids as performed by (Engelhorn et al. 2025).

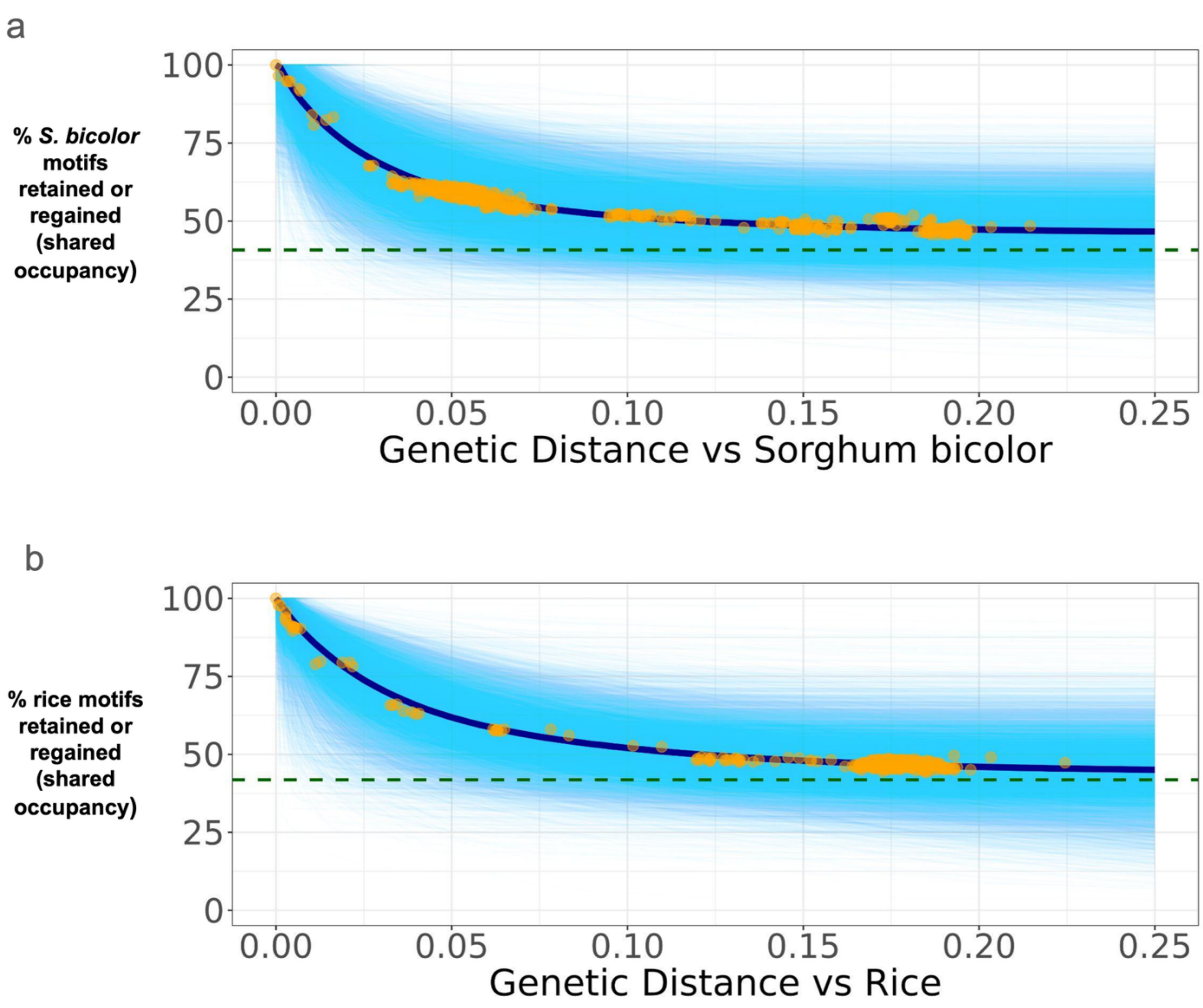

Supplementary Figure 7. Decay in shared motif occupancy relative to *Sorghum bicolor* (a) and *Oryza sativa* (b).
Genetic distance was estimated using pairwise distances between the focal species and the other Poaceae taxa at the Angiosperms353 loci. Blue lines represent exponential decay curves fit for each orthogroup. Orange points show the mean percentage of focal species motifs with shared occupancy conserved in each Poaceae species across all orthogroups, with an exponential decay curve depicted in dark blue. Dashed green line represents the mean percentage of motifs with shared occupancy across 100,000 random pairs of genes in the focal species.

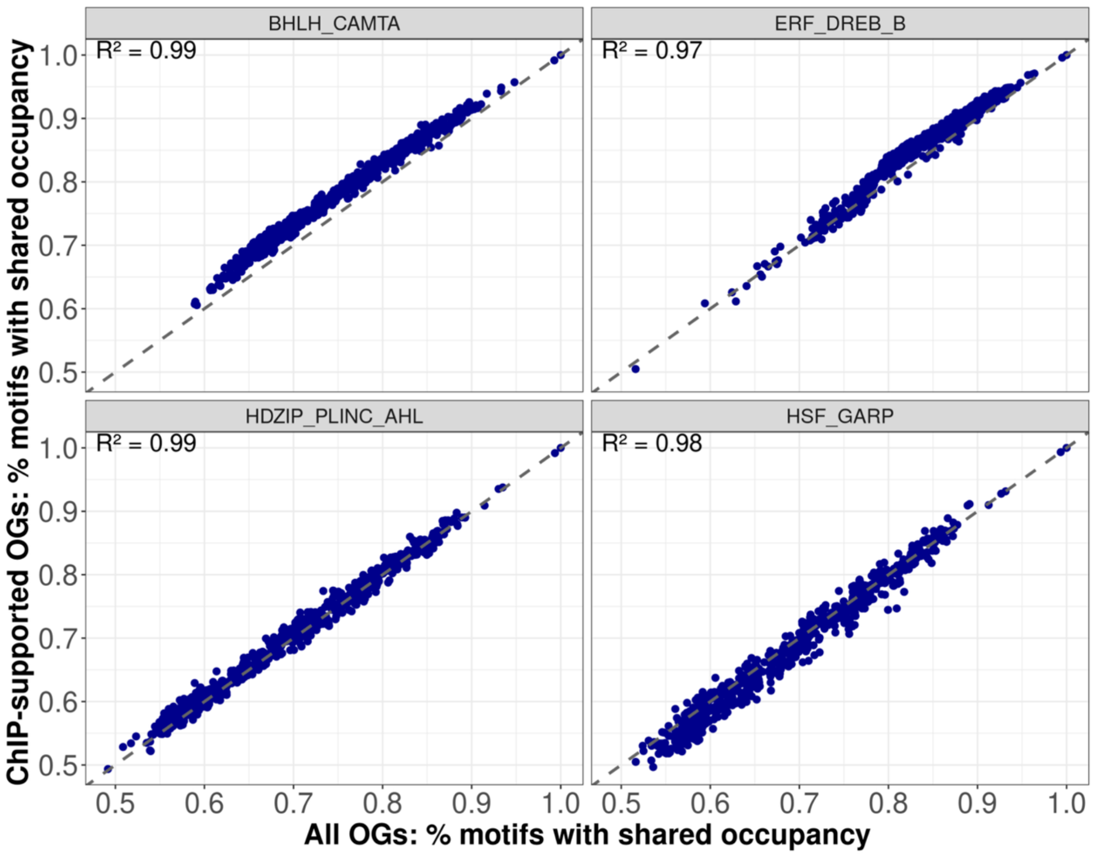

Supplementary Figure 8. Comparison of shared motif occupancy at genes with or without known TF binding evidence.

We compared shared motif occupancy at two sets of orthogroups: 1) The full set of orthogroups that maize was represented in (x axis, "All OGs") 2) Orthogroups with known *in vivo* binding of the focal TF. These OGs have a ChIP-seq peak found within 500bp of the maize translation initiation site (y axis, "ChIP-supported OGs"). ChIP-seq data from four TFs were used to compare shared motif occupancy for four corresponding motif types (bhlh47:BHLH/CAMTA, ereb17:ERF/DREB B, hb34:HDZIP/PLINC/AHL, glk53:HSF/GARP). Shared occupancy for the corresponding motif types is shown. y=x is plotted as a dashed grey line.

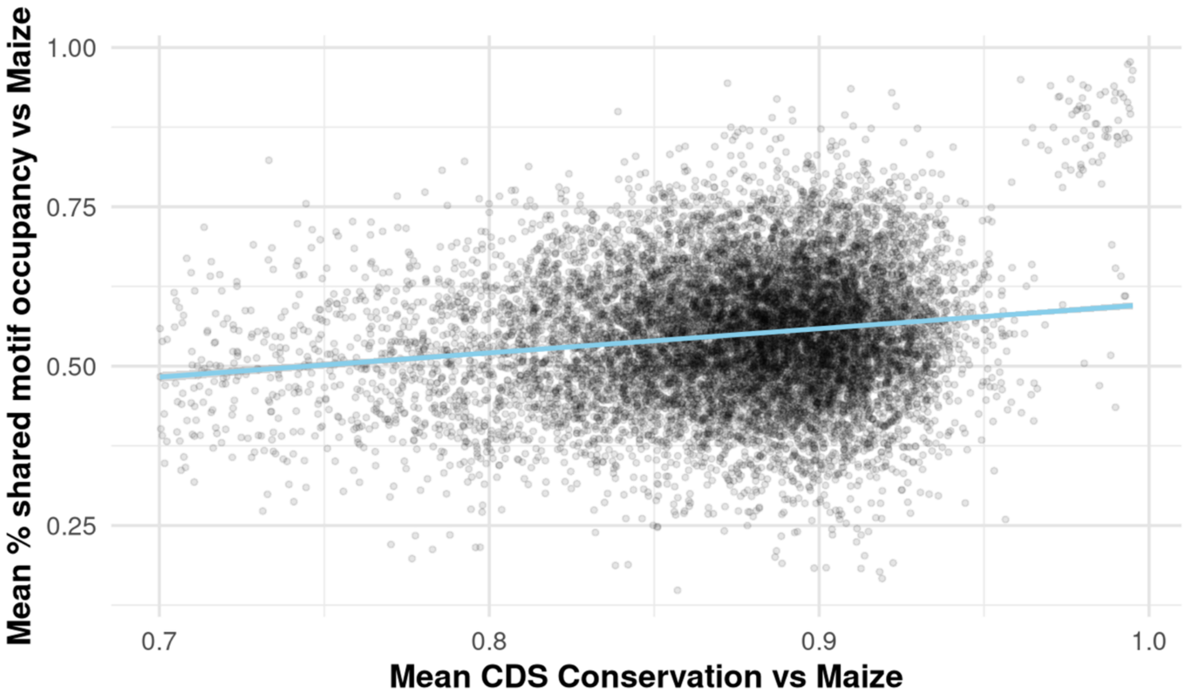

Supplementary Figure 9. Coding sequence conservation vs shared motif occupancy across orthogroups.
For each orthogroup, the mean coding sequence (CDS) conservation and shared motif occupancy values between maize and the 726 other taxa are depicted. A small number of orthogroups with <70% coding sequence conservation were excluded. The blue line depicts a linear model fit to the data with R-squared = 0.02.

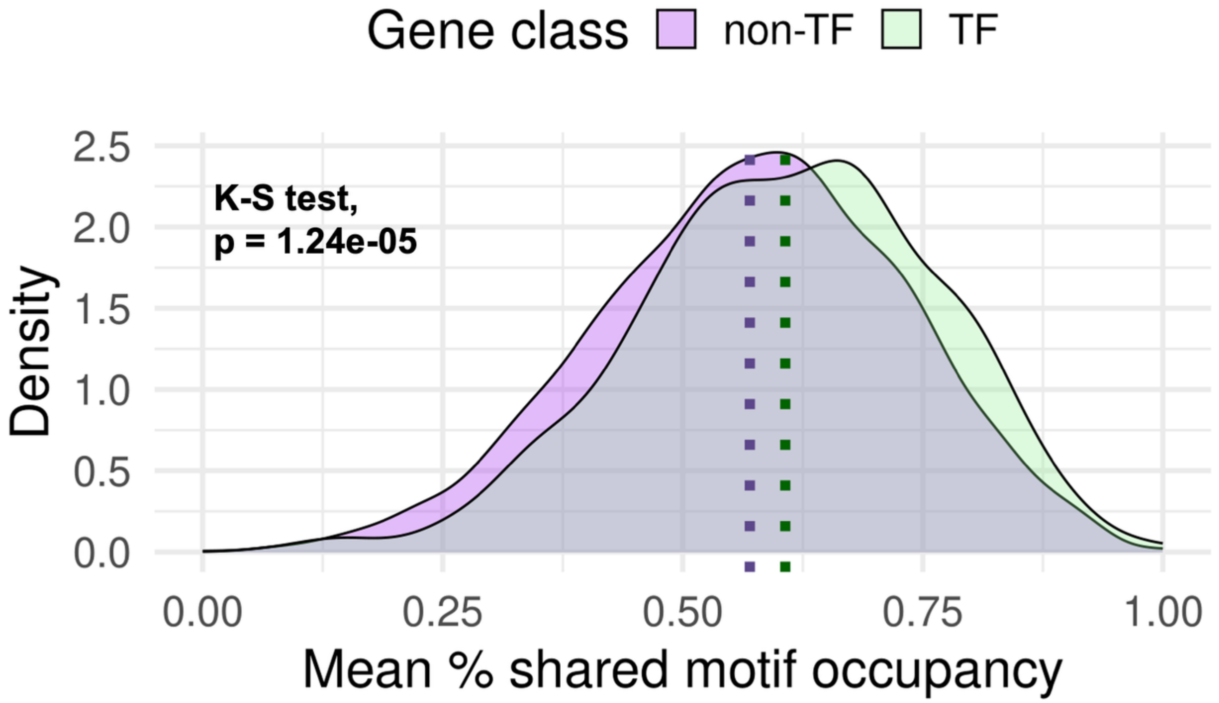

Supplementary Figure 10: Maize vs sorghum motif conservation across syntenic orthologs. Mean % shared motif occupancy is shown calculated across 644 syntenic TF orthologs ("TF") and 5433 background syntenic orthologs ("non-TF").

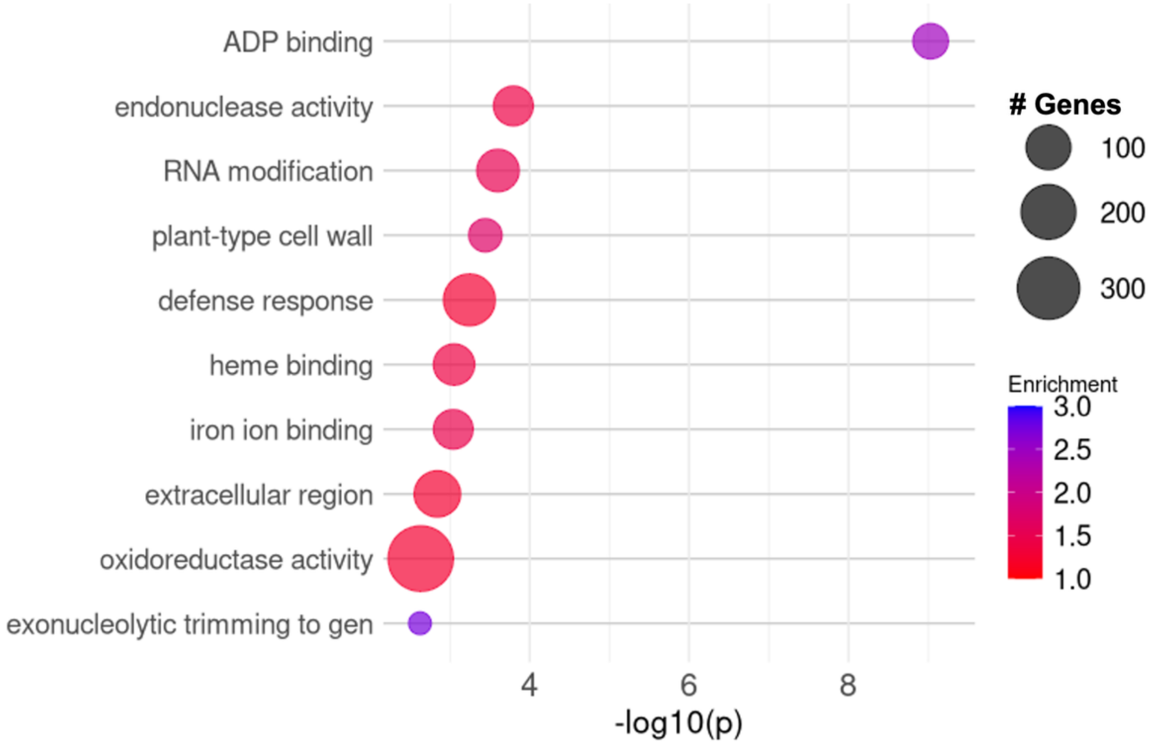

Supplementary Figure 11: Enriched GO terms for orthogroups with low shared motif occupancy across species. Enrichments were calculated for the orthogroups in the bottom quartile of shared occupancy (mean shared occupancy across taxa relative to maize). Bubbles are sized relative to the number of genes associated with each term that are found in the bottom quartile of shared occupancy. Bubbles are colored by the fold enrichment of empirical genes in the bottom quartile relative to expectation.

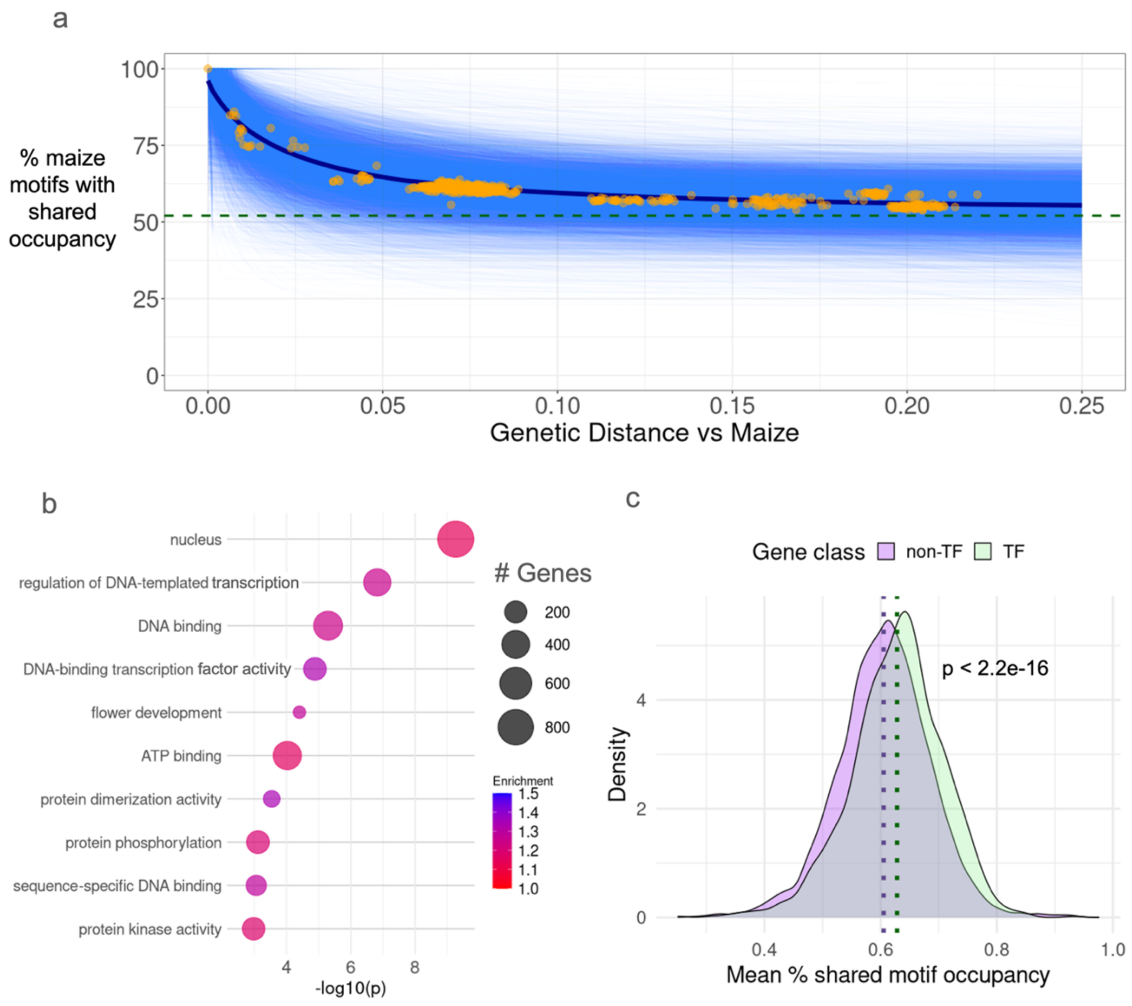

Supplementary Figure 12: Motif turnover shows similar patterns when using 1kb upstream regions.
Motif turnover analyses were re-run using motif occurrences within 1kb of the translation start site. **a.** Percentage of maize motif instances retained or regained (shared occupancy) across 589 Poaceae species. Genetic distance was estimated using pairwise distances between maize and Poaceae species at the Angiosperms353 loci. Solid cyan lines represent exponential decay curves fit for each orthogroups. Points show the mean percentage of maize motifs with shared occupancy in each Poaceae species across all orthogroups, with an exponential decay curve depicted in a dark blue line. Dashed green line represents the mean percentage of motifs with shared occupancy across 100,000 random pairs of maize genes. Approximate divergence times from maize are shown for key taxa (Chen et al. 2022; Gallaher et al. 2022)**. b.** Enriched gene ontology terms for the orthogroups in the top quartile of shared motif occupancy. Bubbles are sized relative to the number of genes associated with each term that are found in the bottom quartile of shared occupancy. Bubbles are colored by the fold enrichment of empirical genes in the bottom quartile relative to expectation. **c.** Shared motif occupancy at transcription factor orthogroups (n = 1,187) versus at non-transcription factor orthogroups (n = 11,385). Median values for each class are shown by dotted vertical lines. P-value is from an asymptotic two-sample Kolmogorov-Smirnov test.

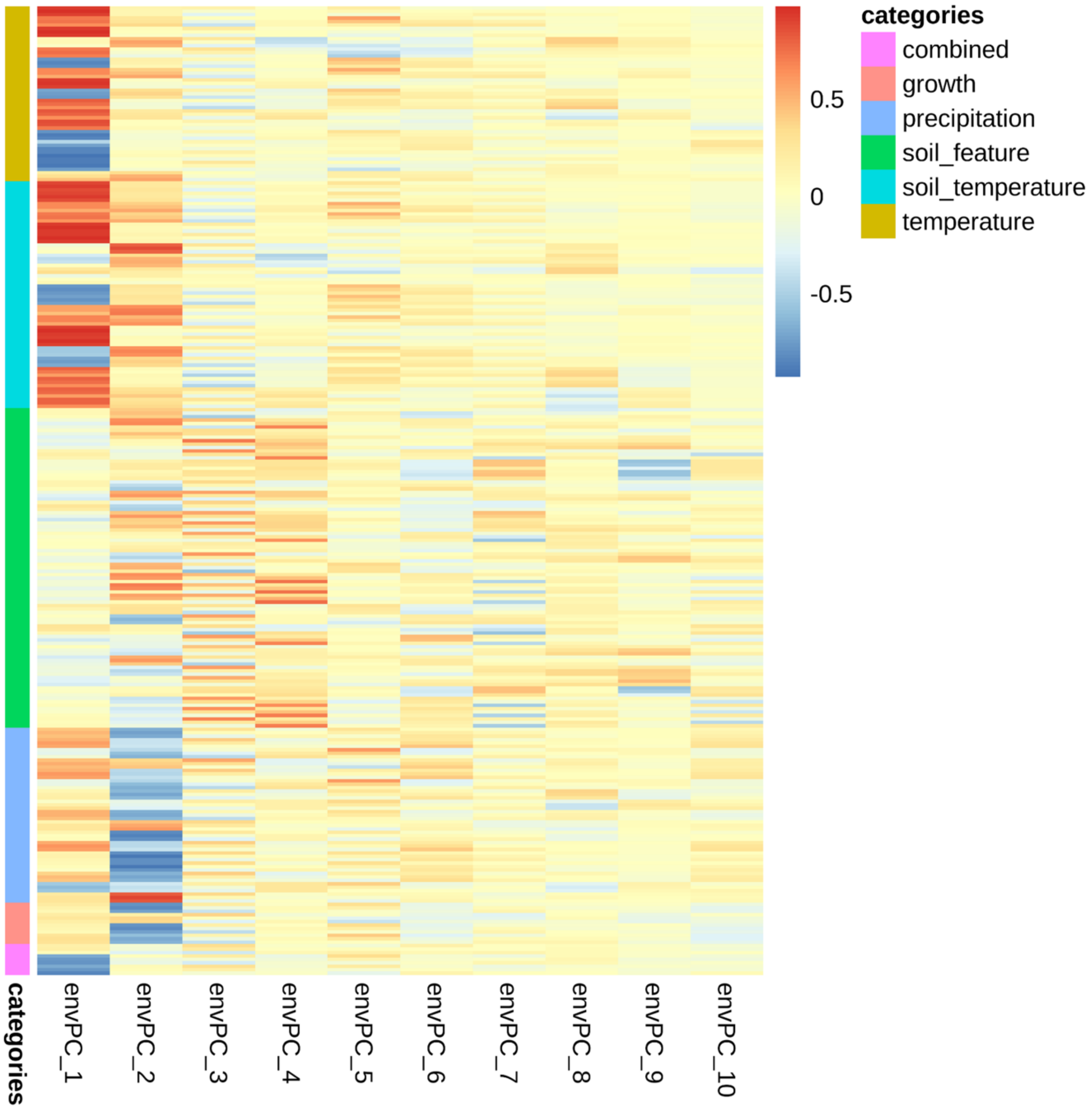

Supplementary Figure 13: Correlation of environmental PCs with individual environmental features.

Individual environmental features were clustered into six categories. Each heatmap cell shows the correlation between the focal environmental PC (envPC) and a single environmental feature.

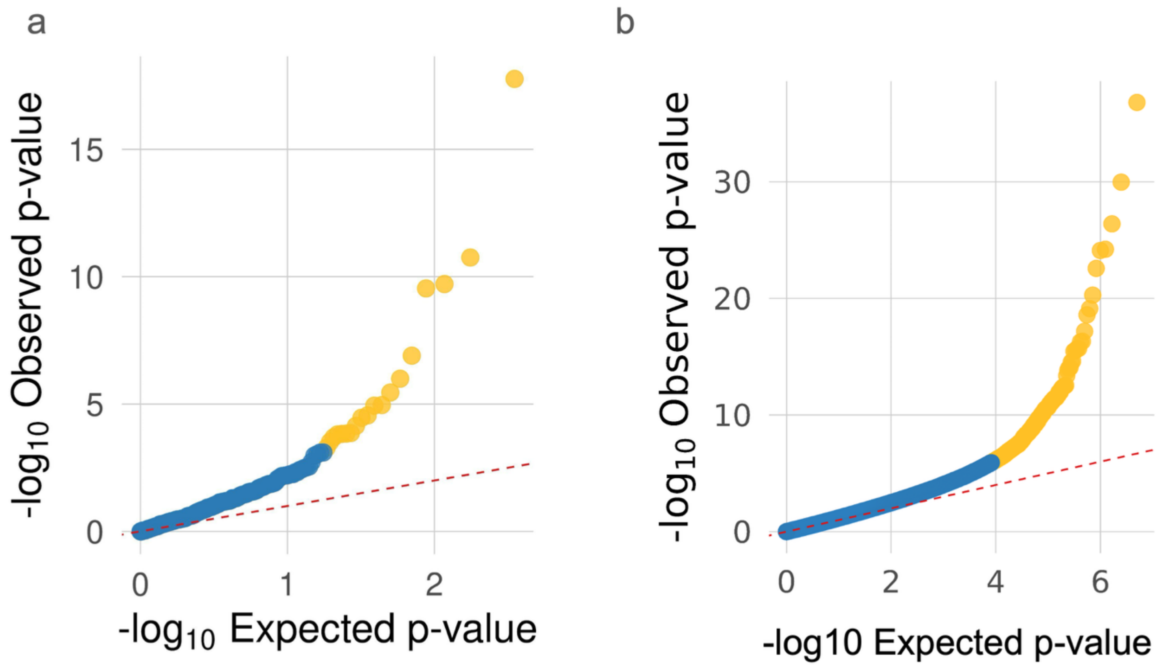

Supplementary Figure 14: Quantile-quantile plots for association models using 1 kb upstream regions for motif scanning.
a) Quantile-quantile plot showing observed p-values from the global motif occurrence models versus p-values expected under the null hypothesis. Occurrence rates were calculated using motif scanning 1 kb upstream of the translation start site. Observed p-values were calculated from Wald tests across 350 fixed effect envPC terms. Terms with FDR-corrected p-values < 0.01 are shown in yellow.

b. Quantile-quantile plot for orthogroup-specific models using 1 kb upstream regions. -log10(p) values are plotted for ~5 million fixed effect envPC terms.

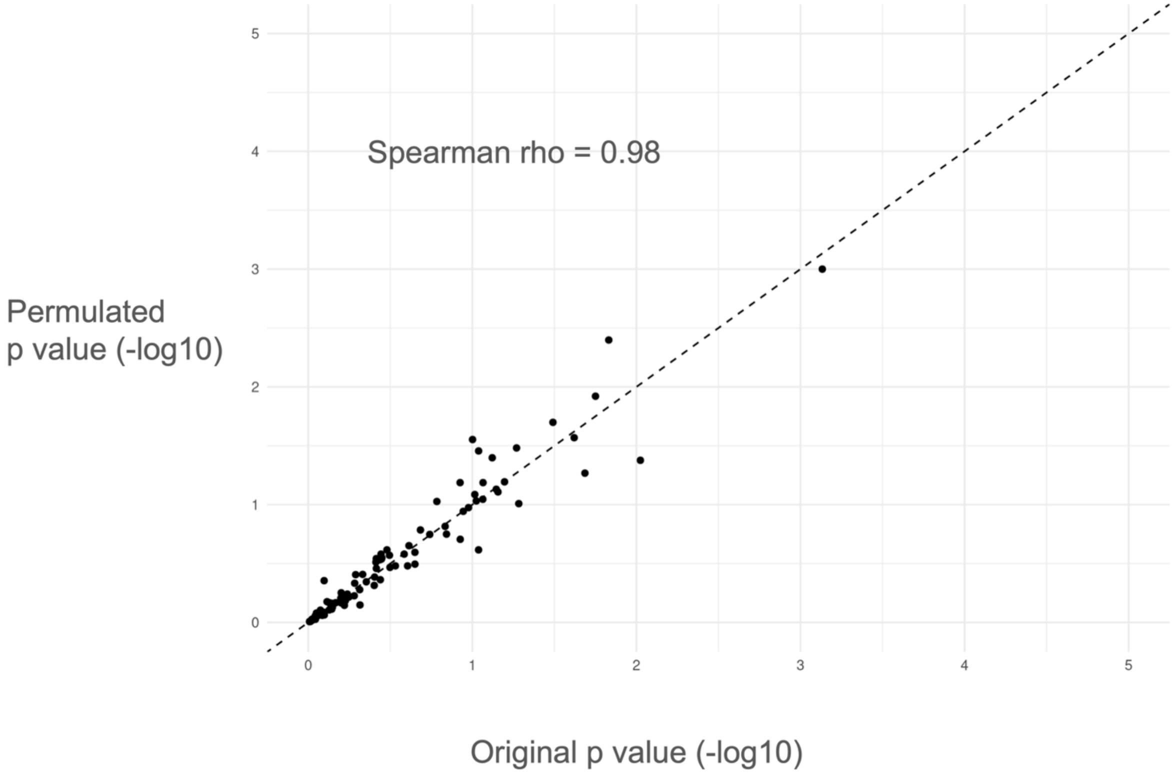

Supplementary Figure 15: Comparison of p values obtained via a standard linear mixed model vs from permulation.
100 motif/orthogroup/envPC combinations were randomly selected for comparison. The focal envPC was permulated 1000 times to create a null distribution of Wald statistics, to which the empirical statistic was compared. A y=x dashed line is shown.
